# Co-Created Solutions for Perinatal Professionals and Childbearing Needs for People with Hypermobile Ehlers-Danlos Syndrome and Hypermobility Spectrum Disorders

**DOI:** 10.3390/ijerph20206955

**Published:** 2023-10-21

**Authors:** Gemma Pearce, Lauren Bell, Paul Magee, Sally Pezaro

**Affiliations:** 1 Research Centre for Healthcare and Communities, Coventry University, Coventry CV1 5FB, UK; lauren.bell@coventry.gov.uk (L.B.); sally.pezaro@coventry.ac.uk (S.P.); 2 Coventry City Council, Coventry CV1 2GN, UK; 3 Centre for Future Transport and Cities, Coventry University, Coventry CV1 5FB, UK; paul.magee@coventry.ac.uk

**Keywords:** maternity, obstetrics, midwifery, anaesthetists, physiotherapy, joint hypermobility, co-creation, co-design, co-production, patient and public involvement

## Abstract

Individuals living with hypermobile Ehlers-Danlos syndrome (hEDS) and Hypermobility Spectrum Disorders (HSD) have reported feeling discredited and unsupported by healthcare professionals. However, the level of knowledge about hEDS/HSD among maternity staff remains unknown. Informed by patient and public involvement, this research aimed to investigate maternity staff’s knowledge and confidence in supporting people with hEDS/HSD, examine people with hEDS/HSD’s experiences of perinatal care, and co-create tools to help maternity staff support people childbearing with hEDS/HSD. Two online mixed-methods international surveys were completed by childbearing people with hEDS/HSD (N = 955) and maternity staff (N = 307). This was followed by the co-creation of three tools with 17 co-creators and a design team. Two main qualitative themes were identified through thematic analysis: (1) a need for recognition of hEDS/HSD in perinatal care and (2) the delivery of appropriate individualised perinatal care. Quantitatively, people with hEDS/HSD perceived maternity professionals to have a low level of knowledge about the conditions. Respectively, maternity staff reported low levels of confidence in supporting people with hEDS/HSD. The co-created tools provide applicable outputs for both education and practice and include an i-learn module hosted by the Royal College of Midwives, a tool for perinatal records, and infomercials.

## 1. Introduction

Standards of maternal and newborn health [1] and intrapartum care [2] state that pregnancy-related complications should be recognised and treated for a positive experience and safe birth. Person-centred perinatal care (inclusive of the postpartum period) for people with long-term conditions is important, with guidance specifically recommending individual assessments be carried out for planning birth with a connective tissue disorder [3], such as hypermobile Ehlers-Danlos syndrome (hEDS) and Hypermobility Spectrum Disorders (HSD). Pre-term birth, pre-eclampsia, eclampsia, pre-term rupture of membranes, antepartum haemorrhage, postpartum haemorrhage, hyperemesis gravidarum, shoulder dystocia, caesarean wound infection, postpartum psychosis, post-traumatic stress disorder, precipitate labour, and being born before arrival at the place of birth were all reported to be higher in those with hEDS/HSD compared to the incidence reported in general populations [4]. While some with hEDS/HSD have relayed positive experiences of childbearing in a qualitative study, many described impaired mobility and delayed recoveries, also impacting their ability to bond and breastfeed their newborn [5]. 

Although hEDS/HSD were previously understood to be rare, more recent evidence suggests these conditions may be more common. In a study of healthcare records, 1 in 500 people were found to have a diagnosis of either EDS or Joint Hypermobility Syndrome (JHS) on their medical records [6], where individuals previously diagnosed with JHS may now be described as having hEDS or HSD. Given that hEDS/HSD are also believed to be underdiagnosed, potential proxy measures [7] of joint hypermobility and chronic pain have been reported with a prevalence of 3.4% [8]. Accounting for population and birth rates, it has then been estimated that 4.6% of all births may be affected by hEDS/HSD [5]. Thus far, the management of hEDS/HSD during the perinatal period can be inadequate due to insufficient awareness of these conditions [9,10,11] and a lack of diagnosis, impeding care and access to appropriate treatment [12]. This can result in perceived hostility and disinterest, creating help-seeking anxiety and clinician-associated traumatisation that can lead to worse but preventable health outcomes [13]. In particular, pregnant people living with hEDS/HSD [5] and parents of children with hEDS/HSD [14] have reported feeling discredited and unsupported by health professionals. Women with hEDS/HSD have perceived that midwives portrayed a sense of panic when trying to support them while lacking knowledge of the conditions [5]. While this qualitative study, which included 40 childbearing women with hEDS/HSD, reported perceptions of inadequate professional knowledge, the actual level of knowledge and understanding about hEDS/HSD among maternity staff remains unknown and would benefit from larger mixed-methods research. By including professionals supporting pregnant people in hEDS/HSD research, new perceptions could indicate how they could be better supported to deliver higher-quality and safer care for those with hEDS/HSD. Additionally, this research not only aimed to identify the potential challenges currently faced in this field but also to develop solutions through the co-creation of tools to help staff support people with hEDS/HSD through their childbearing journey. This is the second part of a programme of research examining childbearing and hEDS/HSD, with the first part examining the incidences of pregnancy and birth complications and outcomes [4].

## 2. Materials and Methods

This research implements a mixed-methods approach [15], combining qualitative and quantitative data in complimentary synthesis, followed by a co-creative methodology to build upon these findings. The research is comprised of three parts: (1) patient and public involvement; (2) two online international surveys; and (3) the co-creation of three tools to support people childbearing with hEDS/HSD. Five third-party organisations (Royal College of Obstetricians and Gynaecologists, The Better Birth Network, Ehlers-Danlos Society, Hypermobility Syndromes Association, and Ehlers-Danlos Support UK) were involved in the development of this project, partners in the funding application, supported recruitment as gatekeepers, and partook in co-creation activities. GRIPP2 was utilised for reporting PPI in this research (Supplementary Table S1) [16]. Previously, hEDS/HSD were named EDS Type III, EDS-Hypermobility Type, and Joint Hypermobility Syndrome [17]. The current study examines and refers to hEDS/HSD, inclusive of these previous diagnoses and nomenclature.

### 2.1. Part 1: Patient and Public Involvement

A poll was created and advertised publicly through the blog of the ‘Academic Midwife’ (www.sallypezaro.wordpress.com) over professional social media accounts. It openly asked members of the public to answer questions about topics and tools important and relevant to childbearing and hEDS/HSD to inform what questions should be included in the surveys (part 2) and potential tools to be co-created (part 3) [18].

### 2.2. Part 2: Online International Surveys

Two online international surveys were completed simultaneously by (1) women with experience of childbearing and a diagnosis of hEDS/HSD and (2) maternity professionals. Quantitative data were collected with additional open-ended qualitative questions to allow participants to clarify their responses. All participants were English-speaking and over the age of 18 years old, residing across the United Kingdom (UK), Ireland, the United States of America (USA), Canada, Australia, or New Zealand. Chosen countries have been included as they are considered developed, and the majority are native English-speaking countries [19]. Inclusion criteria and recruitment of people with childbearing experience are detailed in Pearce et al. [4]. Maternity professionals included any healthcare professionals providing care to childbearing people. Eligibility was open to enable the professional participant group to be multidisciplinary in nature and advertised to a range of professions (for example, midwifery, obstetrics, physiotherapy, anaesthetists, EDS-related specialists, and complimentary therapies). Participants did not need to have experience with or knowledge of hEDS/HSD to participate. All participants were recruited through public, professional, and third-sector platforms. Our recruitment ads were shared >200 times and were viewed by >40,000 social media users. Additionally, paid promotion was utilised on Twitter and Facebook platforms to target a range of healthcare professionals for the staff survey only.

The survey was hosted on Qualtrics software, starting with participant information and consent tick boxes to ensure informed consent was received before participants could complete the survey. Participants with hEDS/HSD completed questions about their experiences in relation to receiving maternity care. Maternity staff were asked questions in relation to their experiences, practices, knowledge, and understandings in relation to the delivery of care to childbearing people with hEDS/HSD. Both groups were asked to reflect on priorities for the development of future research and interventions to support the delivery of safer and higher-quality maternity care for this unique population. A debrief, signposting participants to sources of information and support, was provided. Participants could withdraw by closing the browser at any time, without reason or consequences, and could pause participation by using the ‘Save Later’ option and return to complete the survey within one week. Participants inputted a code of their choice that they could use to withdraw by emailing the researcher up until the point of data analysis. Any identifiable data collected via open-ended responses were anonymised.

For childbearing participants with hEDS/HSD, the Generic Short Patient Experiences Questionnaire (GSPEQ) assessed their experiences of maternity care via a 5-point Likert scale [20]. Responses were changed from ‘clinicians’ to ‘maternity staff’ as the questionnaire was designed to ask questions about staff providing most of the maternity care. Participants also completed questions about their demographics, outcomes, and complications, with these results reported elsewhere [4].

For maternity professionals, descriptive information was gathered about the member of staff’s role and country of residence, along with the length and setting in which they practice. Questions were asked in relation to the members of staff’s awareness, confidence, and decision-making with regards to caring for childbearing people with hEDS/HSD. 

The findings were synthesised using a mixed-methods approach, with quantitative findings used to underpin qualitative themes [21]. Open-ended qualitative responses were analysed inductively using reflexive thematic analysis [22,23]. After authors (G.P. and L.B.) read the data multiple times, semantic codes were created for all the data and grouped collectively for both participant groups into sub-themes and then themes (L.B.). These were collaboratively discussed, reflected, and revised to ensure they were data-driven and defined (with GP) and sense-checked for meaning and relevance to the field of perinatal services (with SP). Illustrative quotations are provided in the prose, with full details of quotations to themes provided in Supplementary Table S2. These are fully anonymised to protect participant identities. Descriptive statistics related to experience are embedded within the qualitative themes. An ANOVA was undertaken to examine whether there was a significant difference in experience of care depending on when a diagnosis of hEDS/HSD was received. An a priori power calculation using G*Power determined a minimum of 200 participants was needed to achieve a power of 0.8 and a medium effect size for each of the surveys. The data collection highly exceeded that minimum, and these data met parametric and homogeneity assumptions.

### 2.3. Part 3: Co-Creation of Tools

Underpinned by participatory action research [24,25,26] and a design thinking approach [27,28], the three co’s framework of co-define, co-design, and co-refine [29,30] was used to identify the best tools to be developed, what should be included in the tools, and refine prototypes towards final outputs. Co-creators were viewed as co-researchers and invited from gatekeeper and researcher networks with experience of hEDS/HSD and/or childbearing, including PPI members, healthcare professionals who support childbearing people, third sector representatives, researchers, and designers (see acknowledgements for list of co-creators). Two online workshops were hosted using Big Blue Button software for co-creators to actively annotate the screen with ideas and engage in the co-creation process. 

The first workshop included co-define activities with an icebreaker to introduce who everyone was and why they wanted to be involved, feedback on the PPI poll, and discussion of what problems needed to be solved and therefore what maternity tools could be created. A consensus was reached on which three tools should be created. These were co-designed together. The core team of Coventry University academics (G.P., S.P., and P.M.) and BenClarkDesign (www.benclarkdesign.co.uk) worked together to create prototypes of the three identified tools. These were presented and discussed for co-refinement in the second workshop and finalised with the five third-sector organisations. 

## 3. Results

### 3.1. Part 1: Patient and Public Involvement

The PPI poll was closed after it received 4000 votes. Those who engaged voted for staff to be asked about awareness, knowledge, and confidence in supporting people childbearing with hEDS/HSD. Therefore, these elements were focused on in the survey (part 2). The top-voted tools to help staff support people childbearing with hEDS/HSD included an online learning module, guidelines, leaflets, a decision-making resource, and an infographic or video. The results of these PPI activities were used to inform the first co-creation workshop (part 3).

### 3.2. Part 2: Online International Surveys

Participants exceeded recruitment aims (N with hEDS/HSD = 955, total pregnancies = 1346; maternity staff N = 307). Most professionals were from the midwifery profession (80%), including 142 midwives, 64 senior midwives, 21 student midwives, 10 consultant midwives, and 9 nurse midwives. A few (7%) were professionals in obstetrics, with eleven consultant obstetricians, five obstetric registrars, three obstetric nurses, and one junior obstetrician. Only 2% were anaesthetists (four consultants and two juniors), and the remaining 11% included physiotherapists, paramedics, osteopaths, chiropractors, paediatric nurses and lactation consultants, a GP, a qualified theatre scrub practitioner, a sonographer, a rheumatologist, and a cardiac nurse. The countries where the professionals were based spanned across the UK (70%), Australia (14%), Canada (4.5%), USA (4.5%), New Zealand (4%), and Ireland (3%). Settings of practice were mainly in obstetric units (44%), community settings (21%), midwifery-led units (7%), specialist clinics (6%), and birth centres (4%), with the remaining settings including antenatal and postnatal clinics, general practice (GP) units, ambulance services, academia, and private care. Descriptive statistics for participants with hEDS/HSD are provided in Pearce et al. [4]. There were two inductively developed themes from the thematic analysis centred on the recognition of hEDS/HSD and the delivery of perinatal care (Table 1). Quotations from participants with hEDS/HSD are accompanied by their diagnosis and country in square brackets, and professionals are indicated by their role and country.

#### 3.2.1. Theme 1: Recognising hEDS/HSD in Perinatal Care

The first theme was about the recognition of hEDS/HSD in perinatal care, with sub-themes on building professional knowledge, developing evidence-based resources, and improving diagnoses of hEDS/HSD. 

##### Subtheme 1: Building Professional Knowledge of hEDS/HSD

Many women reported that professionals often had limited awareness and knowledge of hEDS/HSD: “*I had to tell every nurse I worked with what it was and how it affected me*” *[EDS Type III, UK].* Some had been cared for by knowledgeable specialists or professionals who actively sought to develop their understanding: “*Midwife was not aware of hEDS prior to my treatment, but took the time to research and shared valuable insight with me*” *[hEDS, UK].* Few professionals perceived that hEDS/HSD awareness had recently grown: “*In my hospital, EDS is a new word; people are now mentioning it, but a year ago it was unheard of” [Midwife, UK].* However, professionals also reported that misconceptions towards hEDS/HSD were held by colleagues: “*Many people I have spoken to seem to believe that hypermobility would be an advantage in labour and fail to acknowledge the added pain or tissue damage that could be caused” [Midwife, UK].* Taken together, professionals frequently described wanting more knowledge of hEDS/HSD, including about symptoms and care management: “*More awareness of the condition and associated complications” [Senior Midwife, UK].*

Participants with hEDS/HSD quantitatively reported that they perceived the professionals to have a somewhat low level of knowledge about hEDS/HSD, with maternity support workers, midwives, and obstetric nurses perceived as mainly ‘not at all knowledgeable’ (Table 2). Physiotherapists were viewed as the most knowledgeable, albeit still on the lower end of ‘somewhat’ knowledgeable. None of the professions providing care for childbearing people were rated on the higher end of the scale as mostly or very knowledgeable about hEDS/HSD.

When asked, 36% of staff did not consider themselves to be knowledgeable on hEDS/HSD. Out of those that did consider themselves to have knowledge of hEDS/HSD, the main sources of knowledge were professional (N = 136) and personal (N = 106) experience, followed by reading research (N = 75) and seeing it in the media (N = 48). Very few gained their source of knowledge from university courses (N = 9) or other types of training (N = 22).

##### Subtheme 2: Developing Evidence-Based Guidelines and Accessible Training Resources

Both childbearing women and professionals identified a current lack of relevant training resources: *“I tried to research EDS in pregnancy, and material was hard to come by” [EDS Type III, UK].* Both participant groups also wanted an increase in evidence-based guidelines to inform appropriate care: “*We need more information that will allow the discontinuation of rapidly dissolving sutures in women with EDS*” and the potential link “*with severe vaginal prolapse postpartum” [Consultant Obstetrician, UK].* Professionals wanted a range of resources, including professional guidelines, toolkits, online courses, and posters, though resources should be based on research, “*a study published to provide the advice that I can pass on to the planning/expectant mother*” *[Rheumatologist/EDS Specialist, New Zealand].* It was important that resources were “*easily accessible*”, including in multiple languages, and that professionals had capacity in their roles to learn that *“information is no good if we do not have enough time to access/absorb or implement it” [Midwife, UK].*

Quantitively, when asked what resources professionals would find most useful, the main seven, starting with the highest ranked, were: clinical guidance (N = 254); online course (N = 232); clinical decision-making tree (N = 180); maternity toolkit (N = 143); infographic (N = 130); leaflet (N = 127); and online information sources like webinars, blogs, Twitter feeds, and online health learning boards (N = 97). Other suggestions across four participants included *“professional articles”*, *“access to hEDS/HSD patients and caregivers”*, and *“incorporating into existing resources like the Altered Health Module”.* One participant highlighted the importance of the *“involvement of all major stakeholders”* in the development of resources. This information was fed into part 3 of the co-creation workshops.

Although the request for clinical guidance was the highest, most had not read the published care considerations available [31] (n = 242) because they were not aware of them (n = 207). The survey appeared to act as an invention in itself because some (N = 30) said they read it during the survey itself, and others added a qualitative comment that they would *“read it later”* now that the survey had raised their awareness. For those who had read the care considerations before survey participation (N = 35), they commented that they found it useful for developing a *“better care plan”*, *“discussing it with women to empower them to be the experts and be heard”*, *“sharing with peers”*, being *“aware of the implications of this condition”*, and increasing *“knowledge”* and *“confidence”* to support and create *“individual plans”* with people with hEDS/HSD. One participant was concerned that the care considerations focused on *“risk”* and *“lack of individuality”*, which “*puts pressure on midwives to refer all women on to a high-risk pathway regardless of the woman’s past experiences. It will put fear into midwives when women with hEDS ask for support to birth outside of a consultant-led unit”.* These comments highlight the impact some research in the field has already achieved and may have in the future.

##### Subtheme 3: Improving Diagnosis of hEDS/HSD

The experiences of participants with hEDS/HSD were impacted by whether they had been diagnosed with hEDS/HSD prior to pregnancy or not. Some felt that a diagnosis would have improved appropriate care: “*If I was [diagnosed] we may have been able to take certain precautions” [hEDS, Canada]*. Participants also often reported that, in hindsight, hEDS/HSD symptoms were present during their pregnancy, birth, or postpartum, and some described a worsening of their hEDS/HSD symptoms: “*I experienced my first joint subluxation (hip) in the last trimester of pregnancy and then became very symptomatic in the year after my child was born” [EDS Type III, USA].* Therefore, some with hEDS/HSD suggested that professionals are well-positioned to identify hEDS/HSD in those undiagnosed: “*I saw a number of physios, etc., and not one realised I was hypermobile despite being bed bound for a lot of my 2nd and 3rd trimesters” [JHS, UK].* Some professionals also estimated that they had unknowingly cared for those with hEDS/HSD: *“I have probably looked after women with this condition and was not aware that this was the cause of their symptoms” [Midwife, Australia].*

Overall, most professionals (M years providing care = 13, range 0.5–39 years) highlighted quantitatively that they had heard of some form of hEDS/HSD, EDS Type III, EDS-Hypermobility Type, or Joint Hypermobility Syndrome, with 15% not having heard of any of the listed conditions and 19% having heard of all of them. When asked how many pregnancies they thought were associated with hEDS/HSD each year, most thought it was rare, with either 1 in 20,000 (31%) or 1 in 5000 (42%). 22% thought it was 1 in 500, with only a small percentage thinking it was more common at 1 in 20 (4%) or 1 in 3 (1%).

#### 3.2.2. Theme 2: Delivering Appropriate and Individualised Perinatal Care

The second theme was focused on the delivery of care for childbearing people with hEDS/HSD, with sub-themes on listening and believing what people with hEDS/HSD are saying to professionals, co-ordinating care for these multi-system conditions better across multiple professionals and disciplines, and as a result, improving care and reducing the potentially traumatic experiences that people with hEDS/HSD can have.

##### Subtheme 1: Listening to and Believing Individuals

Collaborative and individual-led care were considered crucial for good perinatal care. Many with hEDS/HSD felt that staff had dismissed their symptoms or not believed their labour progression: “*I gave birth in the car park on my own because they did not believe I was in labour” [hEDS, Ireland].* A lack of trust in these personal bodily experiences sometimes led to increased risks, complications, or traumas, including pain: “*I was still not believed when I said the epidural was not working… I then told them correctly which ones [toes] they touched” [EDS Type III, UK].* Some professionals also highlighted the value of learning from people with hEDS/HSD when co-creating guidelines and care plans, particularly because of the diverse nature of hEDS/HSD: “*EDS presents differently in every woman; communication is key. It is essential to believe women when they report unusual symptoms and not to fob them off because it does not fit in a box” [Senior Midwife, Ireland].* Relatedly, women frequently described having to advocate for themselves amidst a lack of professional understanding of hEDS/HSD. Professionals who had hEDS/HSD themselves often felt better placed to advocate for EDS-informed care: “*I am a registered nurse. I was well researched and advocated for the care I needed” [EDS Type III, USA].*

Overall, quantitative GSPEQ scores [20] for childbearing women’s experiences of care were mixed (Table 3). Tertiles showed an even spread, with a third of participants with hEDS/HSD having a negative experience overall, a third considering their experience to be neither negative nor positive, and a third experiencing positive care. The GSPEQ [20] scored a very high reliability value for this study (α = 0.93). 

##### Subtheme 2: Coordinating Appropriate Perinatal Care for People with hEDS/HSD

Care coordination across professionals and disciplines ensured that information about an individual’s hEDS/HSD was effectively communicated to inform care. Professionals knowledgeable about hEDS/HSD often played an important role: “*The OB [obstetrician] who was a geneticist was a strong advocate for me. She made all the difference with my medical team” [EDS Type III, USA].* However, some participants received care during birth that was not delivered according to care plans: “*My notes about EDS and my birth plan written by the doctors were ignored and led to complications*” *[EDS-Hypermobility Type, UK].* Professionals, often because of a lack of confidence in their understanding, wanted referral pathways to specialists or hEDS/HSD-informed professionals: “*Good signposting when women present with conditions that are outside of our sphere of competence and knowledge*” *[Student Midwife, UK].* Similarly, those childbearing with hEDS/HSD described that other professionals should have been involved in their care, for example, physiotherapy: “*I wish that discharge included a referral for physical therapy” [hEDS, USA]* and mental health teams, “*the maternity carers were completely focused on the baby and not my mental health (due to the immense pain I was in)*” *[JHS, New Zealand].* Restrictions on some health systems were noted. For example, insurance-based systems could limit exploration of health issues beyond pregnancy: “*My insurance was for pregnancy only, so they were unable to address my care even if they wanted to” [hEDS, USA].*

Quantitatively, the professionals most frequently rated feeling “not at all” confident in relation to providing effective care, identifying symptoms, referring, and forming a care plan. Most often, professionals were only “slightly” confident that other colleagues were providing good care (Table 4).

Additionally, there was no significant difference (F_(2,896)_ = 1.12, *p* = 0.33) in experience of care in relation to whether people with hEDS/HSD first conceived before diagnosis (M = 33.84, SD = 9.35), after (M = 34.85, SD = 9.09), or in the same year (M = 33.98, SD = 0.22). 

##### Subtheme 3: Reducing Trauma and Improving Experiences

Participants with hEDS/HSD reported varied experiences that differed across their pregnancies and interactions with different professionals. Positive experiences encompassed compassionate, person-centred, and knowledgeable care: “*The midwives who delivered my baby were extremely calming, welcoming, helpful, and understanding. A couple of midwives in the aftercare made me feel quite silly regarding my hEDS, even though they did not know what it was*” *[hEDS, UK].* Inadequate professional knowledge of hEDS/HSD and inappropriate or harmful care contributed to trauma, anxiety, and depression: “*I am pregnant again and am terrified, have PTSD, and cannot attend the hospital I birthed in*” *[hEDS, UK].* As well as avoiding specific professionals or hospitals, some participants had made birthing choices based on fear of inadequate professional understanding: “*One of the reasons for my second pregnancy is that I am opting for a planned C-section because of how terrified I am of people not knowing how to help me*” *[JHS, UK].* Some had also chosen not to become pregnant again: “*I will never risk pregnancy and childbirth again because women with hEDS are not given the healthcare services they need*” *[hEDS, Australia].* Trauma also contributed to longer-term issues with physical and emotional health and relational problems with infant or partner-bonding; “*as a result of their actions, not only did I almost die, I have never been able to have the relationship with my son I desperately want to have” [EDS Type III, Australia].*

### 3.3. Part 3: Co-Creation of Tools

In the first workshop, three key needs were identified as a focus of the co-creation: (1) a need to educate professionals supporting perinatal care about hEDS/HSD; (2) a need to support shared decision-making between childbearing people with hEDS/HSD and professionals; and (3) a need to raise awareness more generally in the public about hEDS/HSD and childbearing. Although the need for guidelines had also been highlighted, it was acknowledged that published care considerations already existed, so it was more important to raise awareness and educate people about their existence and main messages. A key challenge was identified as wanting to raise the profile by emphasising the importance that professionals understand hEDS/HSD and childbearing, while also not wanting to put people off childbearing with hEDS/HSD. Linked to this was the challenge of explaining hEDS/HSD care considerations as a whole and in a simple, publicly accessible way, while also highlighting the different potential presentations and considerations of supporting people with hEDS/HSD. For example, some people do not respond to local anaesthesia, while others do.

Three tools were decided following discussion. The first was in response to the highest-voted educational tool in the PPI poll, an online learning module, and would address the first identified need of helping professionals learn how to support childbearing people with hEDS/HSD. This could be developed as an accredited and professional i-learn module through the Royal College of Midwives. A prototype short learning course was co-designed with learning objectives, information to be provided, key resources, and signposting. The second agreed output was to address the need for shared decision-making by developing a one-page, freely downloadable tool for perinatal records. A prototype was co-designed to include key information about hEDS/HSD, considerations that maternity professionals and people with hEDS/HSD may want to consider together, and a section to write about aspects unique to that childbearing person and how support could be tailored for them. It features a direct picture from the co-created annotations in Big Blue Button, where one co-creator wrote, “Parents and staff making decisions together”, and other co-creators drew ticks, heart shapes, and ”yes!” around it. The third agreed-upon output was to address the final need of raising public awareness by creating a freely accessible infomercial about hEDS/HSD and childbearing. The idea was co-designed for a person with hEDS/HSD to speak about their experience, a midwife to talk about key care considerations, and then to show them making shared decisions together. BenClarkDesign was commissioned to create the infomercial as part of a team including an animator, voiceover actress, and videographer (see acknowledgement for details).

**Figure 1:**
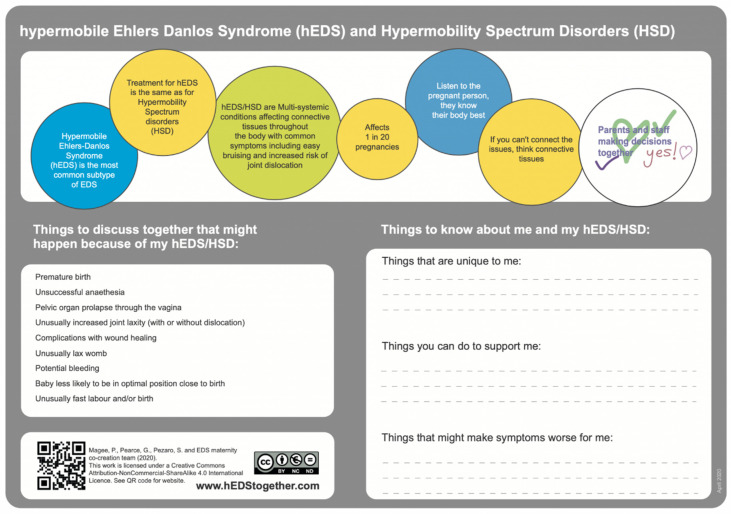
Tool for perinatal records for shared decision-making between people with hEDS/HSD and professionals.

Positive feedback was provided in the co-refine sessions, with co-creators providing annotations directly to the software as well as discussing their thoughts. It was agreed that the tool for perinatal records (Figure 1) would feature in the infomercial (hosted on the Coventry University YouTube channel) and i-learn module (hosted on the Royal College of Midwives website), and the infomercial would be in the i-learn module too. The agreed improvements were applied, and the final versions of the three tools are also hosted on the www.hEDStogether.com research dissemination website. 

## 4. Discussion

Overall, a major qualitative theme identified was the need for recognition of hEDS/HSD in perinatal care through building professional knowledge of hEDS/HSD, developing evidence-based guidelines and accessible training resources, and improving diagnosis for people with hEDS/HSD. Women with hEDS/HSD quantitatively reported that they perceived professionals supporting their perinatal period to have a low level of knowledge about hEDS/HSD with no significant difference in care regardless of diagnosis before, during, or after conception. This is understandable in a context where professionals also reported that they do not feel confident or knowledgeable about the conditions, providing effective care, identifying symptoms, referring, and forming a care plan. Most professionals still consider hEDS/HSD to be rare (1 in 5000 or 20,000), even though it is now estimated to affect 1 in 20 births [5]. They felt this idea of rareness was backed up by the fact that they had not received formal education at university about it and did not believe that they had come across the condition in practice, although because of the survey participation, some reflected that they may have come across people with hEDS/HSD under their care unknowingly. There was a clear need, identified by people with hEDS/HSD and professionals, underpinning the co-creation of educational materials such as the i-learn module and infomercial to guide support through the childbearing process. Some also commented that issues with recognition were exacerbated by the mixture of potential names for the conditions, including older diagnoses or less formal labels like ‘hypermobility’. This corroborates the need identified for a clearer current diagnosis and treatment [12]. In the future and with the use of the co-created tools, the hope is that as understanding improves, more people will be diagnosed, and more professionals will have increased knowledge and confidence on how to support people with hEDS/HSD. 

Qualitatively, the second major theme was around delivering appropriate and individualised perinatal care. The sub-theme relating to a need to be listened to and believed corroborates research across other healthcare provision [11,13,14], with novelty added to the field with the sub-themes about coordinating appropriate care for childbearing people with hEDS/HSD and reducing trauma and improving experiences. It is of note that some professionals who did not know about the conditions commented that they were not aware of potential complications during pregnancy and birth [4] and implications in the postpartum period, such as infant feeding. This corroborates previous qualitative research with people with hEDS/HSD [5] while adding novelty from the professional’s perspective. Additionally, there was a potential miscommunication highlighted where professionals felt they were providing an opportunity for people with hEDS/HSD to explain their condition, but this was interpreted as the need for tedious repetition by multiple professionals with a lack of knowledge. Clearer communication is therefore recommended about the desire to listen and tailor care for the individual, and the co-created tool for perinatal records provides a stimulus for this to occur. Where professionals did have knowledge about the condition and were able to advocate for the person with hEDS/HSD or were prepared to actively research and learn about the condition, this was felt to provide a positive experience. When this was not the case, some reported that it led to complications that could have been avoided. 

There were concerns expressed in both the survey and co-creation that when raising awareness among professionals, the emphasis could focus too much on the risks of the conditions. It was considered important that the co-created outputs and public messaging were not aimed at putting people off childbearing and did not provide the impression that all people with hEDS/HSD are the same and that all should be considered high-risk pregnancies because this is not the case. Although higher risks have been identified with a need for monitoring in cases of a need for higher-risk consultant-led care [4], in some cases, actions taken as a result of being considered to have a high-risk pregnancy could actually cause more harm. For example, serial cervical length screening examining the theory of cervical insufficiency because of abnormal cervical collagen in people with hEDS/HSD has not been shown to decrease the incidence of pre-term birth [32]. However, cervical cerclage placement can increase the risk of tearing in connective tissue disorders [32].

People with hEDS/HSD should not be discouraged from birthing their babies vaginally [33]. Birthing environments that support spontaneous pushing, optimal positioning, and joint support are encouraged to avoid unnecessary trauma to joints and ligaments [31]. Promoting calm and relaxation is highly valuable [33] and helps to naturally stimulate hormones conducive to birth, such as oxytocin and endorphins. Additionally, oxytocin can provide a beneficial antidiuretic effect for people with PoTS [33,34]. Midwife-led care during labour and birth promotes non-pharmacological pain management, resulting in lower medicalisation and intervention [35], especially if the healthcare professional is well-informed about hEDS/HSD. This can help to reduce possible hEDS/HSD-related complications and problematic post-partum symptoms, such as prolapse from episiotomy [36,37], coccyx dislocation [37,38], pubic symphysis diastasis from McRobert’s manoeuvre [39], PTSD [31], and poor wound healing and infection from 3rd/4th degree tears, episiotomy, and caesarean sections [37]. Additionally, potential issues with anaesthesia may be able to be avoided, such as ineffective local anaesthesia [31], issues with cervical spine instability [40], CSF leak risk from epidural [41], and hypotension from anaesthesia [42]. However, for people with PoTS presenting with hypotension-causing peripheral vasodilation, phenylephrine epidural may prevent reactive tachycardia [43]. Precipitate labour, suspected to be due to laxity, meaning easier passage through the pelvis, can reduce the chance of instrumental birth and associated risks [44]. Ideally, for the benefit of both professionals and those childbearing with hEDS/HSD, perinatal staff would be more prepared for this to occur in people with hEDS/HSD and thus better able to avoid births occurring outside of their intended location, potentially with a plan in place for home birth as appropriate or as a contingency [33]. 

### 4.1. Strengths and Limitations

These were large international surveys representing a range of diagnoses related to hEDS/HSD and professions. A high number of people took part, especially in the PPI activities and particularly people with hEDS/HSD, as hundreds participated from the first tweet (<24 h). This highlights a distinct appetite for these conditions to be researched and supported in healthcare. The inclusion of a range of countries provided a depth of experience, often highlighting key similarities in issues such as the need to feel listened to and coordinate maternity care to improve experiences. However, differences in healthcare provision also need to be considered within this context; for example, issues with insurance coverage do not apply across all included countries. Additionally, this was a self-report questionnaire with the potential for sampling bias from those that wish to have their voices and opinions heard about hEDS/HSD, both as people who have had children and perinatal staff. A third of childbearing people with hEDS/HSD reported a negative experience overall, a third neither negative nor positive, and a third experienced positive care. It is worth noting that some participants reported in their qualitative comments that they found this question difficult to answer because they have experienced variation across their care, either within the same pregnancy or across different pregnancies. 

In this self-report questionnaire, sampling bias presents a limitation. For example, healthcare professionals with experience with hEDS/HSD personally or professionally may have been more likely to complete the questions. The recruitment approach aimed to mitigate this by sharing the questionnaire broadly and clarifying that no knowledge or experience of the conditions was necessary to take part. Similarly, one limitation was the relatively low number of participants from some professional groups in the study (e.g., anaesthesiologists), and so the results from these groups cannot be extrapolated to the wider profession’s population. This study used a mixed-methods questionnaire, with qualitative responses used to illustrate and provide completeness to quantitative data. In this way, it is recognised that participant interviews were not implemented to gain in-depth insight from each participant, and the aim of this survey was instead focused on reaching a broader population following previous interview research [5]. The use of a mixed-methods survey approach helped to build upon previously published literature and ensure that the findings could be triangulated and synthesised together in a complimentary way to build up a well-rounded picture, leading to strong co-created outputs. 

Patient and public involvement was important to this research from the beginning, informing focus, design, and outputs. The international vision for PPI identified key PPI barriers, which have been successfully addressed within this research [45]. For example, public awareness and communication were increased through our established networks and the engagement hubs of hEDS Together and ‘the academic midwife’ [18], co-creators were acknowledged where consent was provided, consistency was increased through the use of the three co’s framework [29], and the authorship team included those with lived experience of hEDS/HSD and of childbearing/parenthood, as well as being multidisciplinary across health psychology, midwifery, and design. The co-creation not only included patients and the public but also other key stakeholders that could provide barriers or facilitate the success of the outputs [29], including third-sector organisations, healthcare professionals, academics, and designers. The online and creative nature of the co-creation workshops helped provide a neutral environment where everyone’s voice was considered equal and inclusive for those who could not attend in person due to disability, time, or travel constraints [29]. In this article, the terms that were used during participant recruitment have been included for consistency (for example, women and maternity). However, we are grateful to the people who contacted us during the study, highlighting that this language excludes trans and non-binary people who have given birth. We have responded to this and used alternate inclusive language (for example, perinatal) where appropriate. Although many of these experiences may be transferable to the trans and non-binary communities with hEDS/HSD (for example, not being listened to and professional lack of awareness), we acknowledge that because the language of ‘women’ was used in recruitment, their experiences will not be fully represented here. Additional research was conducted to consider perinatal care for trans and non-binary people separately [46].

### 4.2. Implications

The three co-created tools provide highly applicable outputs for use in both education and practice and could help to raise the profile of the current published care considerations [31,33]. However, further work needs to be carried out to raise the profile of the developing evidence base further into guidelines, such as with the Ehlers-Danlos Society and the National Institute for Health and Care Excellence. These would need to incorporate the additional knowledge from this current paper and the linked publication on outcomes and complications [4]. The developed i-learn module for members of the Royal College of Midwives can now also be expanded on a larger scale, for example, into university curriculum for trainee professionals and a publicly accessible Massive Online Learning Course (MOOC) for Continued Professional Development (CPD) of healthcare professionals caring for childbearing people. Further research would be useful to objectively examine cohorts of childbearing people to more accurately assess the prevalence of hEDS/HSD accessing these services and more fully understand how perinatal staff and childbearing people can work together to increase the positive experience and safety sought by World Health Organisation policy [1,2]. This could specifically include further examination of the use of Vicryl rapide and increased severe postpartum vaginal prolapse observed by one of the consultants who participated in this research. There is also the potential for training and evaluating the use of specialist hEDS/HSD midwives as front-line givers of care and hubs of knowledge for other perinatal professionals, like the current models of diabetes and epilepsy care, for example.

## 5. Conclusions

This mixed-methods research is the first to examine professionals’ knowledge and confidence to support childbearing people with hEDS/HSD and is complementary to a large international survey examining the experiences of childbearing with hEDS/HSD. Not only was the research informed by PPI to ensure the focus was meaningful, but solutions to the identified problems were then co-created to provide applicable outputs to education and practice. The online CPD module is hosted officially by the Royal College of Midwives’ i-learn platform for its members, and the tool for perinatal records and infomercials is freely and publicly accessible to support shared decision-making and raise awareness.

## Figures and Tables

**Table 1:** Outline of themes and sub-themes identified from the thematic analysis.

Themes	Sub-Themes
Theme 1: Recognising hEDS/HSD in perinatal care	Subtheme 1: Building professional knowledge of hEDS/HSD
	Subtheme 2: Developing evidence-based guidelines and accessible training resources
	Subtheme 3: Improving diagnosis of hEDS/HSD
Theme 2: Delivering appropriate and individualised perinatal care	Subtheme 1: Listening to and believing individuals
	Subtheme 2: Coordinating appropriate perinatal care for people with hEDS/HSD
	Subtheme 3: Reducing trauma and improving experiences

**Table 2:** How knowledgeable each maternity professional was perceived to be about hEDS/HSD (in order of most knowledgeable to least).

Knowledge (Scaled from 1 to 4, with 1 Being Not at All Knowledgeable and 4 Being Very Knowledgeable)	Mean (SD)	95% CI
Physiotherapists	2.21 (1.00)	2.12, 2.29
Obstetricians	1.81 (0.90)	1.73, 1.88
Anaesthetists	1.81 (0.96)	1.73, 1.90
General practitioners (GPs)	1.54 (0.67)	1.49, 1.59
Obstetric nurses	1.40 (0.70)	1.34, 1.46
Midwives	1.38 (0.69)	1.32, 1.43
Maternity support workers	1.21 (0.53)	1.16, 1.25

**Table 3:** Descriptive statistics for each item of the GSPEQ.

Experience (Scaled from 1 to 5, with 1 Being Very Negative and 5 Being Very Positive)	Mean (SD)	95% CI
Easy to understand	3.78 (1.04)	3.71, 3.85
Confident	3.58 (1.15)	3.50, 3.65
Informed	3.37 (1.19)	3.29, 3.45
Suitable	3.07 (1.28)	2.98, 3.15
Involved	3.52 (1.21)	3.44, 3.60
Organised	3.29 (1.16)	3.21, 3.37
Appropriate	3.45 (1.24)	3.37, 3.53
Satisfactory	3.39 (1.22)	3.31, 3.47
Benefited	3.56 (1.13)	3.48, 3.63
Correct treatment	3.15 (1.23)	3.07, 3.24
Total (scaled from 10 to 50)	34.16 (9.31)	33.55, 34.77

**Table 4:** Maternity staff confidence levels to provide care to people with hEDS/HSD (%).

How Confident Would You Be to:	Not at All	Slightly	Moderately	Very
Provide effective care	37	27	27	9
Identify symptoms	54	25	14	7
Refer	31	25	22	22
Form a care plan	32	24	27	17
Colleagues provide good care	28	37	25	10

## Data Availability

Raw data is not publicly available due to ethical restrictions, but anonymised and themed qualitative data is provided as a supplementary file to this publication. The corresponding author can be emailed with questions about the data. Publicly accessible information can be found about the programme of research on this website (www.hEDStogether.com).

## Supplementary Materials

The following supporting information can be downloaded at: https://www.mdpi.com/article/10.3390/ijerph20206955/s1. Table S1: Summary of PPI using the GriPP2-SF Reporting Guidelines; Table S2: Quotations underpinning subthemes from thematic analysis.
