# Supplementary material for: Co-Created Solutions for Perinatal Professionals and Childbearing Needs for People with Hypermobile Ehlers-Danlos Syndrome and Hypermobility Spectrum Disorders
Source: Int J Environ Res Public Health. 2023 Oct 21;20(20):6955. doi: 10.3390/ijerph20206955 (PMC10606217; doi:10.3390/ijerph20206955)
Supplement: Supplementary file 1 [file ijerph-20-06955-s001.zip › Supplementary file 1 PPI summary using GRIPP2 SF.pdf]

Supplementary file 1

Table S1. Summary of PPI using the GRiPP2-SF Reporting Guidelines

Items	Description of how items were addressed
1: Aim	To collaboratively involve patient and public members in the design of the research and the co-creation of the tools to inform perinatal education and practice.
2: Methods	A poll was created and advertised publicly through the blog of the 'Academic Midwife' (www.sallypezaro.wordpress.com) over professional social media accounts [16]. It openly asked members of the public to answer questions about topics and tools important and relevant to childbearing and hEDS/HSD to inform what questions should be included in the surveys (part 2) and potential tools to be co-created (part 3)<br><br>PPI members were involved in the co-creation team and viewed as co-researchers. Two online workshops were hosted using Big Blue Button software for co-creators to actively annotate the screen with ideas and engage in the co-creation process. The first workshop included co-define activities with an ice breaker to introduce who everyone was and why they wanted to be involved, feedback on the PPI poll and discussion of what problems needed to be solved, and therefore what maternity tools could be created. Consensus was reached on which three tools should be created. These were co-designed together. The core team of Coventry University academics (GP, SP & PM) alongside BenClarkDesign (www.benclarkdesign.co.uk/) worked together to create prototypes of the three identified tools. These were presented and discussed for co-refinement in the second workshop and finalised with the five third-sector organisations.
3: Study results	The PPI poll was closed after it received 4000 votes. Results of these PPI activities were used to inform the survey questions (part 2) and the first co-creation workshop (part 3).<br><br>PPI members were part of the co-creation team, who decided the needs needed to be addressed, discussed the challenges being faced, decided the three tools to prioritise creating, co-designed the three tools, and provided feedback on prototypes of the three tools for their co-refinement.
4: Discussion and conclusions	PPI members provided important insight that helped design the research and the outcomes being co-created. Their input also helped to frame the discussion sections, such as caution about focusing on the risks only potentially putting people off of becoming pregnant with hEDS/HSD or worrying perinatal professionals supporting them.<br><br>Informal PPI feedback was also provided over social media from trans and non-binary people regarding the language of 'women' in recruitment, and so the language in the paper was changed where appropriate to focus more on 'childbearing people', and 'perinatal' rather than 'maternity' care. A separate research study was also conducted following this feedback to consider perinatal care for trans and non-binary people [44].
5: Reflections/critical perspective	Patient and Public Involvement was important to this research from the beginning informing focus, design and outputs. The international vision for PPI identified key PPI barriers, which have been successfully addressed within this research [43]. For example, public awareness and communication were increased through our established networks and the engagement hubs of hEDS Together and 'the academic

midwife' [16], co-creators are acknowledged where consent was provided, consistency was increased through the use of the three co's framework [27] and the authorship team includes those with lived experience of hEDS/HSD and of childbearing / parenthood, as well as being multidisciplinary across health psychology, midwifery and design. The co-creation not only included patients and public, but also other key stakeholders that could provide barriers or facilitate the success of the outputs [27], including third sector organisations, healthcare professionals, academics, and designers. The online and creative nature of the co-creation workshops helped provide a neutral environment where everyone's voice was considered equal, and inclusive for those who could not attend in person, such as disability, time or travel constraints [27].
