# Supplementary material for: Co-Created Solutions for Perinatal Professionals and Childbearing Needs for People with Hypermobile Ehlers-Danlos Syndrome and Hypermobility Spectrum Disorders
Source: Int J Environ Res Public Health. 2023 Oct 21;20(20):6955. doi: 10.3390/ijerph20206955 (PMC10606217; doi:10.3390/ijerph20206955)
Supplement: Supplementary file 1 [file ijerph-20-06955-s001.zip › Supplementary file 2 quotations from qualitative analysis.pdf]

Supplementary file 2

Table S2. Quotations underpinning subthemes from thematic analysis.

**Theme 1: Recognising hEDS/HSD in perinatal care**	
Subtheme 1: Building professional knowledge of hEDS/HSD	
Women with hEDS/HSD	
	I've seen [an anesthesiologist] this time that knew about my condition and gave me a lot of advice about what drugs and situations are best for someone like me I.e., epidurals wouldn't be advised and if I had to have a c-section it would be best to use a spinal block.
	The obstetrician was very knowledgeable and I felt comfortable in her care.
	The antenatal community midwife knew nothing about EDS and had no interest.
	Staff was not knowledgeable about EDS.
	Community mw was inconsiderate of my condition and umbrella'd *[sic]* any symptoms on being pregnant.
	The healthcare providers had no understanding of hypermobility or the impact of pregnancy on this… There was no understanding or good enough care/support/awareness for hypermobility issues.
	Anaesthetist was planning to go do some research.
	They did their best to educate themselves about EDS and take precautions.
	The knowledge of the practitioners varied immensely […] midwives who didn't seem to have any idea of what my limitations or issues might be.
	Anaesthetists as part maternity care I feel need more education on the lack of mobility of mothers in pain with pelvis back and hips etc on top of their labour pain.
	My doctors were aware of my EDS and careful to watch for and prevent haemorrhage.
	My first one was fairly rubbish in that they all thought I was type 1.
	The obstetricians were very keen to research about EDS although did not seem to know much about it.
	Always dismissed as normal as no one new of hypermobility type problems.
	Nobody had a clue about my needs.
	No one was knowledgeable to help my situation.
	Nobody had much experience if any of HMS
	Our team didn't do any reading on my diagnosis and admitted knowing nothing about it. Their expectations were ludicrous and completely ridiculous demands were made of me considering my hypermobility.
	I told them I probably have hEDS but hadn't been diagnosed. They said they had other patients give birth with no issues.

	They didn't seem to fully understand about hypermobility.
	Every professional told us there was no longer a link between hypermobility (known at the time but not labelled HSD) and precipitate labour.
	I gave up mentioning EDS (was suspected for long time before diagnosis) medical staff make you feel like you have Munchausen's so in the end I stop taking about it.
	Scoffed at my mentioning of EDS.
	I found my condition to be dismissed and not seen as much to consider.
	I really don't think they knew anything about EDS or autoimmune disease, but then neither did I at that time.
	I hope more OBGYNs, midwives, and maternal health specialists become informed about hEDS so women like me have better, more supportive birth experiences.
	Health professionals who understand hEDS would have made a great deal of difference in my recovery.
	I did not know I had a syndrome at the time, but had my doctors been more knowledgeable I believe it could have gone much smoother.
	Better understanding and general midwife training is urgent.
	They are becoming more knowledgeable, but nobody had a clue how to treat me during both of my pregnancies.
	I think going forward everyone needs educated.
	I wish more obstetricians were aware of the benefits of waterbirth for those with unstable pelvis rather than rushing to offer c-section as only option.
	It's challenging because during my pregnancy every medical professional I dealt with hadn't had a patient with hEDS before. Every stage was new difficulties and things that couldn't be explained to me.
	I would happily welcome a change in educating the medical profession on how to better support women with hEDS
	I really wish more doctors, nurses, midwives and medical professionals would take the time to read through the paper.
	They were good but because they knew nothing about EDS explaining it every time the midwives swapped shifts was very annoying
	It is so nice to know that Midwives and Doctors are pursuing a better understanding of hEDS/HSD.
	No empathy from NHS Doctors with regards to EDS most never heard of condition. Was told no natural birth from knee / hip surgeon due to my hip dysplasia but they still made me give birth naturally and now have told me I'm in a mess...Sadly for me I watched them google my condition.

	None of my maternity care came from practitioners who were already well versed in EDS, but some really went out of their way to educate themselves and provide excellent care. One only admitted their lack of knowledge after unintentionally dislocating my hip when they refused to listen to me, but that led to them doing more research and becoming one of the best people on my team […] more than one told me they'd never heard of EDS and suspected it wasn't real.
	I believe health care professionals need educating in HMS and symptoms. I am still struggling to find GPs that understand and are willing to help with referrals. We are not taken seriously.
	The midwife at the hospital did not know about hypermobility syndrome and did not believe me when I had a very rapid labour.
	I generally found midwives did not understand my condition, nor appreciate my knowledge of it.
	I gave birth at [UK hospital] which holds itself out as knowledgeable on EDS. Whilst they know about the sensitivities and extra considerations, when it came to the day to give birth they ignored it all. The actual doctors on the labour ward did not have a clue about EDS.
	My care team, led by an excellent Consultant Obstetrician, understood my condition and managed it well.
	No one seems to understand Ehlers-Danlos and all the ways that it affects the entire body
	No knowledge from staff regarding the condition. No link noted between Ehlers-Danlos, rapid birth and tearing/pelvic floor. Did receive advice once I had requested it and pushed for it.
	I found I knew more about my condition and unfortunately spent most my time explaining it rather that dealing with my issues during pregnancy.
	I would say very few of them knew much about hypermobility or really how to respond when I tried to inform them I had it.
	If my midwife had been better informed she may have been able to recognise the symptoms and been more sympathetic and able to advise me better.
	No-one knew a single thing about hypermobility syndrome, I didn't know the implications either!
	HSD understanding needs a really big push in the medical community. No one knows what it is and the few who know about EDS refuse to believe it can be as serious/debilitating as EDS.
	Midwife in most recent birth was aware of hEDS and researched herself personally to help with best care plan
	My prenatal care was really excellent. The team were knowledgeable and I was encouraged to make plans appropriate to my condition
	I was high risk and my maternal fetal medicine specialist was so amazing! She knew all about EDS. The normal OBs at the hospital were not as knowledgeable
	No-one really understood EDS

	People didn't really know anything about EDS
	No one knew anything about EDS
	Although my midwife did not know about my EDS, she […] read up on it before my following appointment.
	The midwives were very helpful given how they did not have a lot of knowledge about EDS.
	No one seemed to hear hypermobility it was just fibro.
	Second pregnancy my obstetrician was quite knowledgeable as he had a Dr friend who was a HSD specialist
	Just wish EDS gets better looked at and recognised more. Lack of knowledge from doctors is sorrying *[sic]* and frustrating
	They were good but didn't know any potential complications of eds and mcad [Mast Cell Activation Disease]....i.e anaesthesia not working.
	I feel that although due care and knowledge was given to pain in relation to my JHS, medical staff did not seem aware or explain other possible complications such as slow healing and issues with anaesthesia.
	My midwife had never even heard of it. If she hasn't heard of it how can she look after me adequately?
	Only 1 person knew what my condition was and I had 2 breakdowns in front of doctors because they did not know anything.
	None of my care team had ever encountered HMS.
	My physicians weren't familiar with EDS
	I feel now that I have my diagnosis there are very little doctors in this country that have any idea about hEDS.
	Please promote the education of maternity nurses on soft tissue conditions like EDS, none of my practitioners even knew how to spell it let alone how to deal with it. I had to tell every nurse I worked with what it was and how it affected me prior to treatment, and it was a huge source of anxiety for me during an already difficult and uncomfortable pregnancy.
	No knowledge of EDS.
	The biggest issue for me was the lack of knowledge regarding how quickly EDS patients can go through the anaesthesia.
	The hospital staff knew nothing of hyper mobility or Ehlers-Danlos (not diagnosed then) and before, during and after even now
	No one knew a thing about HEDS
	Many midwives didn't understand my disability
	I gave birth alone because the staff didn't recognize how close to delivery I was.
	They didn't have a clue about the condition they just felt I was flexible
	I feel the lack of understanding of Hypermobility EDS was a big negative impact.

	There needs to be better understanding of PoTS symptoms during labour
	Hospital was totally clueless about EDS very little support or advice
	Even my maternal fetal medicine physician had a very poor understanding of hypermobile EDS.
	Condition was on notes as hypermobility only and was not taken seriously.
	No one had considered the potential of poor wound healing before, during or after my C-Section. No one had considered that my child may have inherited hEDS.
	No one really knew about EDS and the implications it would have on my body
	None of the staff I saw had even heard of EDS so I was in charge of telling THEM how to care for me, which didn't give me a huge amount of confidence. I would have thought that after telling the lead midwife that I had it that they would be professionally bound to look up information on it so they knew how to care for me
	Although most of the maternity services I encountered had to ask me what EDS was by the next appointment (for those with whom I had appointments) they had thoroughly researched as much as they could
	My Maternal Fetal Medicine doctor was the most knowledgeable about Ehlers-Danlos and took the necessary precautions.
	Midwife was not aware of hEDS prior to my treatment, but took the time to research and shared valuable insight with me for discussion.
	It was only the chief of medicine in the fetal maternal ward that knew anything about EDS and the care I needed to receive.
	I only hope this research will lead to maternity staff being more educated for others.
	In 2007 I was told by my attending midwife that EDS was 'an old wife's tale' and every woman had problems through pregnancy
	My OB was unfamiliar with EDS, but did a lot of research on his own and trusted my knowledge on the subject.
	My OB was great the second time around, and actually researched my condition, so he could make the best plan for me and my baby, but everyone else besides him, knew next to nothing about EDS
	The last minute obstetrician made extra appointments to get to know me and was well aware of her lack of knowledge about EDS, so she got knowledge. Same with the anaesthetist, they researched the best drugs to use for the spinal. I was very impressed both knew the limits of their knowledge.
	I found the knowledge of maternity health care professionals on PGP [Pelvic Girdle Pain] varied greatly, I was very fortunate to have a midwife who understood the condition and pushed for me to receive the right support and treatment.
	My obstetrics team and head anaesthetist were very knowledgeable

	Initially nobody talked to me about the potential risks involved in pregnancy with my EDS type 3 diagnosis, I became concerned with this and raised it with the community midwife. [I shared the latest research article with my midwife and this was passed to my consultant and added to the front of my notes. Subsequently I felt my care improved]
Maternity professionals	
	More awareness of condition and associated complications
	That whole awareness of multiple disorders that are often quite rare. We never stop learning as diagnostic testing reveals ever increasing dysfunctions and disorders or is this mutations to find the new normal?
	More information on the whole as have come across this but usually obstetric team who plan the care.
	More awareness and discussion.
	Ensuring other providers are knowledgeable. I.e., nurses, midwives, nurse anaesthetists
	Cause and affects on their birth experiences. How to help ease their symptoms
	Signs & Symptoms
	what the conditions are, symptoms, complications antenatally, intrapartum and postnatally, care planning and appropriate referrals
	What they are more at risk of and ways to decrease these risks.
	Basic information about what it is and how it can affect pregnancy and how I can support women throughout pregnancy and beyond
	Basic information about the condition and the impact on pregnancy including what referrals would be appropriate
	Awareness and signposting
	About symptoms, how this may impact on maternity and how we can best support women
	Type of referrals needed. More information on condition.
	Having a deeper understanding of how the condition can affect childbearing in all aspects. Having an awareness of an individual's condition when booking for pregnancy so that any choices can be discussed fully and the woman can be supported to make informed choices and reduce the fear amongst staff that something has been missed.
	Not very relevant to me as a specialist infant feeding midwife, but I am always interested to know more about anything to do with pregnancy and childbirth. I would expect women with the condition to be able to educate me, if they have received good care themselves and understand their condition. So info from women will eventually educate midwives.
	This has highlighted my lack of knowledge about EDS.

More information needs to be shared widely.
Knowing more about the condition, it's effects on the woman & baby and how to provide the best support
Increased awareness of less common conditions that may affect women in pregnancy and childbirth.
In my hospital EDS is a new word, people are now mentioning it, but a year ago it was unheard of. I print the guidelines every time I see a patient with EDS.
I have no knowledge of hEDS/HSD
Common issues to watch out for in each patient regardless of type of EDS - joints, pain management, SPD, mental health etc. Information about red flags - cardiac/vascular issues that might render a patient high risk, needing specialist involvement/referral etc.
Basic knowledge of symptoms/lived experience
Background information on hEDS
Many people I have spoken to seem to believe that hypermobility would be an advantage in labour and fail to acknowledge the added pain or tissue damage that could be caused. The current lack of awareness could mean sub optimal care when minimal changes could vastly improve a birth experience
Any information. A general overview of the details midwives need to know, actions needed and risks to be aware of so the safest most appropriate care can be planned for the individual woman
An understanding of the condition(s) in question and knowledge of how pregnancy, childbirth and post-partum recovery are affected.
How the condition affects the woman in relation to life and childbearing.
Advised care pathway. Red flags to be aware of.
The effects of hEDS on the non-pregnant and pregnant woman, how this can have an effect on her during the antenatal, intrapartum and postnatal period. If there would be any effects of the hEDS on the newborn.
Specific disease presentation management and effects of and on pregnancy and birth
Physiological impact on pregnancy
More knowledge on symptoms and effect they have on the patients.
More general information with numbers affected, evidence-based care and complications
Different types and how that affects gestation
Key points to care. Risks and things to avoid
Just signs and symptoms and how to provide care. Risks for mum and baby
Information on the condition itself, plus what this can mean for women at different stages of the perinatal journey; information about how midwives can positively work with women with the condition.
General information as to the effect and management of EDS

	Doctors might not know much about it either. I've encountered many who've no idea, including my own GP
	How EDS and associated conditions can impact pregnancy, how to individualise care with each woman, what best practice points should guide care, what to normalise v what’s not normal.
	More awareness around the condition
	Education on the condition, it’s prevalence, symptoms and management.
	Guidance around best way to give birth, guidance around physical safety in labour, guidance around antenatal care etc,
	Greater education for the wider team, a lot of HCP [healthcare professional] don’t recognise the associated risks
	Symptoms and issues that can worsen in pregnancy. Things we need to monitor and include in a woman’s care plan antenatal, intrapartum and postnatally
	How it effects women. Symptoms. Pregnancy related problems that could occur
	How it can affect women generally and during the childbearing period
	How these conditions effect pregnancy and how pregnancy effects these conditions. I think with this information any competent midwife, along with the MDT, can help these women make informed decisions on their care and birth.
	We need the topic highlighted again, so it stays in people's minds.
	Thank you for making me aware of these syndromes
	Not very relevant to me as a specialist infant feeding midwife, but I am always interested to know more about anything to do with pregnancy and childbirth.
	Needs more knowledge about hEDS/HSD
	Named link midwife in each maternity unit would be a good start to increase awareness
	More awareness is definitely needed
	Knowledge to midwives is power we can give to the women
	I would welcome more relevant information so I can effectively care for my patients
	I will read about more information to provide better clinical care for women with this condition
	I think education is really important. It is good to educate all health care professionals.
	I have only a vague knowledge of hEDS/HSD and mostly because I'm borderline HSD myself.
	I have hEDS […] Raising awareness would really help so that people might at least have heard of it, might help with things like being listened to so that you get adequate pain relief, or physio referral etc.
	I find it amazing that I had not heard of this syndrome before

	I feel very passionately about educating professionals on EDS but find it very difficult because people have many misconceptions they believe to be true and it is very difficult to change attitudes
	I feel lithotomy should be contraindicated with EDS and there is overall poor understanding of this. I feel medical staff should be made more aware of EDS and the challenges it can present in pregnancy
	Generally it is poorly understood, I think, especially in relation to adaptations/adjustments in pregnancy, labour and p/n period
	As much as I have heard of hEDs, it has only been in the recent few months that it has been highlighted by women in the media, and so I have proactively started to educate myself
	I haven't come across anyone in my 13 years who has EDS so considering care hasn't even entered my head […] But it would be interesting to learn about the effects of this condition on women who do become pregnant.
Subtheme 2: Developing evidence-based guidelines and accessible training resources	
Women with hEDS/HSD	
	Midwives and hospital maternity workers need to be trained in our condition and how it can affect us.
	Staff in hospitals need training.
	Staff often contradicted each other as to what was best
	I tried to research EDS in pregnancy and material was hard come by. Doctors in the UK should have mandatory training on EDS.
	I was told by numerous doctors that my pre-term pregnancy was random and that there is no information supporting why it happens. Although I do believe some cases can just happen, I do believe we need to look at the preterm labor rates as being higher amongst women with hEDS (at least what I have gathered from forums, seems to be a group of us)
	Because of not much information about HEDS no one is able to give me a straight answer of what is wrong with me
	I asked a variety of health professionals (GP/Midwife/consultant) how hypermobility may affect me and my pregnancy. None were able to say or offer any advice. I was not aware what pelvic floor exercises would be protecting me from. I feel I was at greater risk of a prolapse and no one told me.
	Staff were not able to inform me of the benefits/risks of natural birth or C-section as far as my JHS was concerned.
	Why can they not simply DEVELOP their practice and insight and understand how hypermobility syndrome affects us?

	Although my pregnancy was well looked after there was little to no care for pain in joints and no one know much about how to help me.
	She [midwife] was unaware prior and wasn't able to get any info internally
	Midwifery staff and obstetric staff followed printed guidance that my mum supplied taken from large up to date book on EDS. They used these printed pages throughout and had no protocol or knowledge in place apart from these print offs
	More needs to be done to train midwives about it and hospital staff.
	Two separate members of staff told me they didn't know how I was going to give birth and my physio said she thought it would be hard for me to do so without dislocations and I felt was not sufficiently knowledgeable
Maternity professionals	
	There is no information out there in practise about hEDS
	A general knowledge base is good, but resources for more in-depth discussion would be necessary for me to take on or consult with this type of patient.
	The best resource would be a trust policy as that is what most clinicians will refer to first and will help to standardise care. The second-best resource could be a leaflet or toolkit that the woman with EDS carries, she could share this with each provider she meets, allowing her to control discussion about her care. This may also be helpful to those less proactive clinicians who have not sought out extra learning about EDS or who have not seen any mooc [Massive Online Open Course]/blog/awareness campaign that is offered
	Clear information leaflets/website with information directed at healthcare professionals not patients
	We need more resources and to get more understanding out there. I feel like one person fighting a battle trying to get the message out. We really do need a tool kit and more resources.
	Any education from: on site inservices from our in-house midwifery educators, to leaflet to online course to video and/ or pod cast ...would be helpful
	As I have never come across this condition in practice, and it is not incorporated into any uni modules, I have not taken the time to read up about it.
	Never remember learning this in my training or even being aware of looking after any women with this condition
	NICE or local guideline/protocol
	Clinical practice guideline. But, it would depend on who wrote the guideline - if it was medically-driven or based on principles of physiological birth. So, I would want to know more, but have space to consider how to best support a birthing person as a midwife as well.
	As much info as possible by any means. Online learning modules would suit me best.

	An easy-to-read awareness graphic of the issues that hypermobility disorders and EDS can cause
	Workshops online training
	Evidence based
	We need more information which will allow the discontinuation of rapidly dissolving sutures in women with EDS which leads to a breakdown in healed tissues causing more prolapse and a difficulty in repairing due to the tissues not supporting appropriately due to the collagen issue
	Toolkits that can be used on an individual basis. Online course to increase knowledge and awareness
	The GP toolkit is helpful
	Symptom management for midwives, clinical supports and decision-making tools and a clinical guideline would be super helpful
	Study days, online social media coverage and posters
	Snappy but to the point concise information. In a busy clinic setting, it can't be difficult to sift through wordy text. An infographic or simple visual care pathway would certainly be better
	Simple guideline: [what the conditions are, symptoms, complications antenatally, intrapartum and postnatally, care planning and appropriate referrals]
	Summary of research in terms of antenatal care, birth preferences, postnatal choices
	Session during study week and online training
	RCOG Stratog module/ e-learning module
	Quick and easy to read.
	Proper clinical guideline
	Online training packages are helpful (who doesn't love a certificate!), I also find infographics really useful to get a lot of info quickly
	Online leaflets, online presentations etc.
	Online course via RCM
	One source with peer review clinical guidelines
	Module to learn about it
	Any information about how best to care for these women would be appreciated in a learning module online
	Leaflet summarising what it is, symptoms, implications for childbearing and care management guidelines summarised.
	Having best care advice easily accessible
	Guidelines, Protocols, Clinical management, study days
	Guidelines

	Guideline/flow chart
	Guideline and patient info.
	General clinical info as a start
	For it to be added to study days.
	Evidence-based...but I suspect insufficient studies exist in this area to give anything other than general observational data
	Evidence-based pathway
	Education in a well prepared and universally understandable format
	Easily accessible, brief but thorough online information. E.g. webinar recording, presentation online, or flow sheet/decision tree.
	Easily accessible online.
	Details on care pathways and information on how to find knowledgeable consultants
	Decision-making trees that reflect relative risk factors rather than absolute cut off points.
	Database of resources, workshops, educated staff sharing their knowledge.
	Clinical tree
	clinical guidelines; care plans
	Clinical guidelines. Access to wide range of resources.
	Clinical guidelines that are easily searchable through GTI clinical guidance - not just by heading but by key words. Sometimes it can be difficult to find the information I need without opening several guidelines and then performing searches for key words.
	Clear guidelines on what it is and how to best support women. What to look for. What to escalate
	Care pathway, clinical guideline, online education modules
	Better training within trusts and better communication within the MDT [Multi-Disciplinary Team]
	A clinical guideline would be extremely useful.
	A toolkit would be great
	A study published to provide the advice that I can pass on to the planning/expectant mother so that she can provide it to her obstetrician/midwife etc.
	A study day and guidelines
	A study day
	A poster which can be read with a pathway that can be followed at a glance
	A guideline to look up when you meet these women. A learning module is not helpful as you need the info when women present, not at 'some distant time when you did that module 6 months ago'.

	A crib sheet possibly for easy access or information.
	a clinical guideline and associated patient information sheet
	A book
	When there's a condition like this that affects a reasonable amount of women, having it included as a session in mandatory training, or at our monthly meetings. A clear flow chart or guideline is also useful so you can look up what you actually need to do with women with a condition, rather than just referring to the consultant
	Information that is easy to remember and points towards the right places to look for evidence-based advice and help - I don't think courses and modules for an infrequently seen condition would have good uptake but easy to recall symptom information and guidelines / leaflets that gave more specific advice would be helpful.
	Free, easily accessible information. Collaboration with professional special interest groups such as POGP for pelvic health physiotherapists to raise awareness of resources once available.
	Clinical guidelines
	Clinical guideline based on evidence-based research to help assist my decision making on areas I do not have as much knowledge about.
	Inservice lead by a medical officer always is informative and raises awareness
	Information on where to access info and especially what is available in other languages
	Information leaflet to give pre conceptually, at rev & pregnancy confirmation with GP, booking appt. with midwife, intrapartum & postnatal leaflet.
	Information is no good if we don't have enough time to access/absorb it, or to implement it (if they need anything 'extra')
	Info sessions at relevant events such as professional conferences, CPD modules, articles, leaflets, in-services
	In service session on EDS/HSD would be an effective way to learn. And to have a CPG on labour Care, referral pathways etc.
	I think all staff should receive training modules across all grades and professionals, official guidelines would be helpful also
	I prefer 20 minute videos or easy to understand article with options and cases
	We really need a tool kit
	I, myself, have EDS, there is a distinct lack of training regarding it, and it’s comorbidity of POTS, which I also have. There is no training, or guideline at my university or trust.
	More training on this will be very useful. I am excited someone will be coming to talk to us about this

	I think that the most important aspect for conditions such as this is to have a robust/sound website to serve as a resource. When I don't know much about particular conditions I will look for charity/support group/specific websites to see if they have info for 'health professionals' and then specific info that is related to pregnancy/labour/postnatal periods so I can quickly get up to speed on the condition which then informs my care delivery.
	I think an infographic identifying symptoms/signs of suspected/subclinical ED would be helpful
	I've never had a pregnant patient with either condition (to my knowledge) and never had any teaching on the subject. I would find it invaluable.
	I have learnt about EDS via my contact with patients in the preterm birth clinic. There is little consolidated information and it would be useful to have something - perhaps an RCOG guideline or SIP?
	I feel that my department and staff would best engage with a poster which could be put up. We have lots of emails with online learning which we don't have time of shift to look at so they get ignored. Managers are happy with posters being put up because it's no effort for them but easy for staff to look at frequently and learn slowly.
	Have encountered quite a few women with some kind of hypermobility in my practice (triage midwife in large urban hospital) but never had any teaching or training about this condition
Subtheme 3: Improving diagnosis of hEDS/HSD	
Women with hEDS/HSD	
	My pregnancy also kick-started more issues with my EDS particularly with my pelvis and lower spine.
	I was unaware that I had hEDS at the time of my labour and suspect this would have made a significant difference to my experience
	This was prior to my EDS diagnosis although I had family history of which I discussed with the Drs… Giving birth has been considered my trigger point for my autoimmune crash according to my immunologists.
	My chronic pain and inability to walk during pregnancy was brushed off as part of pregnancy. No one looked further into it
	Although I wasn't diagnosed at the time of my 1st 3 pregnancies I now know why some of my problems were worse than those of other pregnant people I knew
	I am currently pregnant and now older my symptoms related to my hypomotility are much worse, but because I'm aware of why they are happening I am able to make the right allowances
	[I told them I probably have hEDS but hadn't been diagnosed. They said they had other patients give birth with no issues.] I was diagnosed 4 months after giving birth
	Was unaware and my doctor was unaware of my diagnosis.

Whilst I had no formal EDS diagnosis I presented with obvious symptoms and was waiting to see consultants.
My 1st pregnancy was difficult and I was not diagnosed with JHS at this time, no one could explain why my hips would sublux.
Diagnostic awareness needs to be increased to ensure women aren't facing serious healthcare complications without adequate medical and rehabilitation support services.
I was not diagnosed with either of my pregnancies however it was known to various professionals (with my 2009 birth) that I was hypermobile due to previous dislocations.
I also hope it becomes easier for women to be diagnosed so they have access to care.
I was undiagnosed with EDS during both my pregnancies, and developed severe pelvic pain and incapacity by 15 weeks in both instances, and had to use a walking stick... None of the practitioners I met during either pregnancy spotted that I was hypermobile, let alone that I might have Ehlers-Danlos...I remain angry at all the lost opportunities to identify my underlying health conditions
I was not diagnosed with hEDS at the time, though showed every symptom
My HSD was not diagnosed at this time and therefore not raised with maternity medical staff although problems with pain and nausea raised and previous problems with anaesthetic raised
I was not diagnosed with hEDS when I was pregnant, I was diagnosed after in large part to my rapid decline during my second pregnancy and since.
I did not have a hEDS diagnosis during the pregnancy and I think there were numerous features which could have led to diagnosis by midwives, physiotherapists or obstetricians, especially given the experience of my previous other pregnancy.
I was not aware of hEDS at the time of my pregnancies. My doctors never suggested looking further into any issues or pains.
Not one maternity-based healthcare professional noticed my hypermobile Joints
I think maternity staff should be amongst the many members of the medical profession who are seeking out information about patients who may not know they have HSD or related conditions. There were many signs I had it, from my life in general, so it's possible that it could have been picked up earlier if it had been looked for.
My OB was a highly respected doctor who specialized in high risk cases. I hadn't been diagnosed with hEDS at the time and she didn't recognize any of the symptoms or offer any help.
At time of pregnancy and post-natal, although I experienced physical indications of EDS, I was unaware and undiagnosed with this condition & it was not a consideration during my maternity care.
All the signs have been there but no one connected the dots

	I saw a number of physios etc and not one realised I was hyper mobile despite being bed bound for a lot of my 2nd and 3rd trimester
	The hospital staff failed to notice the dislocation and various GP appointments and physio nothing was discovered.
	Had issues in both of them and so many things pointing to hEDS yet nobody picked it up or ever mentioned it
	Maternity Physiotherapist completely missed my hypermobility and EDS, wrongly diagnosed with PGP
	With one hospital it was during my second pregnancy that I was diagnosed thanks to the OBGYN who during my first appointment saw "hypermobility" on my chart and referred me to the head geneticist at their hospital who was able to diagnose my EDS.
	I was slightly symptomatic, but undiagnosed during this first pregnancy and childbirth. I experienced my first joint subluxation (hip) in the last trimester of pregnancy and then became very symptomatic in the year after my child was born, which lead to a diagnosis about 2 years after my child was born
	I think midwives are in a good position to spot previously undiagnosed hypermobility conditions and should be encouraged to look for further signs if symptoms such as SPD, carpel tunnel etc. are presenting in a patient.
	I wish I had known I had EDS and all that that entailed about early delivery, haemorrhaging, pelvic dysfunction risk of pre-eclampsia, etc
	I didn't know I had hEDS at the time I gave birth. I wish I had. I would have known to ask for a lot of different kinds of support.
	I was not diagnosed with hEDS at the time of my pregnancies. If I was we may have been able to take certain precautions.
	If I had been diagnosed with EDS prior to my son and daughter being born I strongly believe that the pregnancies would have not been so traumatic.
	An earlier diagnosis […] would have made a great deal of difference in my recovery
	My biggest regret was not being diagnosed with POTS which put me and my child at risk as I was continually collapsing and nearly fell down the stairs at least once.
	I wasn't diagnosed hEDS until after all of my children were born and I think it would have helped tremendously in providing better medical and emotional care throughout.
	The only one I'm lucky to have is my gynae who has helped me to get my diagnosis
	My youngest son showed signs of HMs as a baby when they did the hip test and the midwife said it would sort itself out. Obviously it didn't.
Maternity professionals	

	In my health visiting role I have referred children to paediatric colleagues and parents to rheumatology who I suspect have hypermobility/EDS (again mostly based on my own knowledge and experience) and they have been diagnosed as a result. If health professionals had more knowledge of the condition/associated conditions this should be a common place occurrence rather than rarity.
	I suspect the diagnosis is simply not considered and so remains hidden
	I guess with hindsight I have probably looked after women with this condition and was not aware that this was the cause of their symptoms.
	HMS so underdiagnosed in the general population, and so frequently attributed to relaxin
	As both a Pediatric and mother provider (lactation support), I wish that more patients were diagnosed. I see newborns monthly that I figure will reach criteria when they are older - they feel different, which is how I knew from day one literally that my kids were EDS despite no one ever being diagnosed or even investigated in my family.
**Theme 2: Delivering appropriate and individualised perinatal care**	
Subtheme 1: Listening to and believing individuals	
Women with hEDS/HSD	
	Nobody listened to me or believed me
	They wouldn't listen to me saying baby was coming, wouldn't let me birth naturally.
	No one listened to me. They had policies and followed them regardless of my history. I told them that once my water broke the baby would arrive in minutes. They refused to call the Doctor until I was completely dilated. Then the baby was coming and they were yelling at me that I had to wait for the doctor- as if I could stop the baby from coming out.
	Staff never respected my assertion of EDS and it's requirements.
	No one understood what was happening and they treated me horribly - like I didn't know my own body.
	My life is permanently damaged by my wishes being ignored.
	The ones in the main hospital had no clue and didn't wish to listen to my opinion or what I had to tell them… they didn't believe me when I said I would probably have baby quickly
	Midwife would not listen baby was coming super quick and didn't care to look. Hated that midwife, she was rubbish, soon started panicking when she actually read my notes and baby was coming!
	[My midwife…] told me that I needed to just get on with things when I tried to explain the exhaustion, pain and loose feeling I had in all my joints.
	No respected any of my wishes. No one listened to me. They took control of me and pushed me around at the hospital.

	Hips dislocated during childbirth and no one understanding why I was screaming in pain they thought it was just contractions
	When the midwife stitched me up she refused to believe the local hadn't worked.
	Was asking for pain relief and was told I didnt need it. I was screaming I needed to push and was told I didnt as was only 6cm. I went from 6cm to baby born in 5mins
	I was not diagnosed at that point, so was treated as if I was 'making up' notions of dislocations
	I gave birth in the car park on my own because they didn't believe I was in labour. My labour went from 0-60, the staff were in complete shock. If only they would believe that people like me birth quickly!
	Both were born from start to finish in 12 hours after water breaking. It was pretty damn easy, which is why with my first nobody believed me I was in labour. Second one was a much nicer experience, as people listened to me this time.
	Told them I could feel it, they didn't believe me at first and said gentle tugging feeling was normal, it wasn't until I started vomiting from the pain that they put me under general anaesthetic.
	I went from 1 to 10 inches within an hour and was not believed by the midwife until someone else checked
	After the pregnancy the pain continued, I had a hard time getting doctors to take my pain seriously, they just kept assuming I was depressed and being dramatic
	The doctor said he'd put anaesthetic in, so it must be working - it wasn't working. I now know why this will have been happening. He should have known that it doesn't work on all people and he should have believed what I told him.
	I had to initiate pushing alone in the assessment ward as I was ignored for being over dramatic, husband was told to go home and told that I would still be in labour the next day. Luckily he didn't leave.
	I wish doctors were more proactive regarding common EDS joint issues especially back and hips. I wish they would not just disregard our pain as "normal".
	I was in agony my 3rd trimester. My doctors blew it off and I think they just thought I was whining. I started walking with a cain, and they were always confused as to why at every appointment
	My concerns about an epidural were mostly ignored and they kept trying to push it on me which I refused.
	Family history of both [hypermobility and precipitate labour]. Constantly told we were wrong and I wouldn’t have one - I did have a precipitate labour
	They were casual and dismissive of my concerns, and brushed off my feeling that something was not right
	Even when in a lot of pain I was treated as just a first time pregnancy and making a big deal. My labour was very intense and quick and midwives were slow to respond to me. I nearly gave birth in a lift as I was dismissed as making a fuss.

More needs to be done to listen to EDS sufferers who understand their condition and their own bodies.
Medical workers need to be aware that patients with high pain tolerances may not find labour that difficult.
The doctors didn't believe me and told me I had no clue what I was talking about despite having already had 4 other kids.
I lost feelings in my right leg in early labour and staff were not understanding that I wasn't then able to walk
I made the staff aware that the epidural had not worked and I wasn't believed and refused further pain medication during labour. Staff only believed I could feel everything when they tried to stitch the 2nd degree tear.
Was not believed when I expressed to staff that epidural was not working
During labour the staff did not believe I was fully in labour as wasn’t screaming with pain. I live in pain so pretty used to it.
Staff argued with me and insisted I wasn't in labour until I insisted they examined me, I was then told immediately to push as they could already see the head
I was clearly in distress and I wasn't taken seriously and nothing was looked into. At a home visit after birth the midwife told me to get up and I'd feel better, when in fact my stitches were infected, I had mastitis and was developing Sepsis
They didnt take me seriously during my first labour, the pain meds did nothing to help and they just assumed it was "normal" pain when I was suffering badly from my pelvis being beyond weak and separating.
Nobody believed me
Did end up with injuries that no one had believed me on up until 2017 when they finally decided to listen and look, then confirm
Everyone thought I was exaggerating.
Symptoms were largely ignored and asked if I was dramatic (passed out while driving).
Midwives told me all the pain was normal. No it's not normal to feel your hips moving apart at week 24.
Because of the lack of the maternity unit listening to me when I arrived just under 2 hours before giving birth, I nearly gave birth in the cafeteria. I ended up birthing in the booking in area
With that said, my concerns regarding pain relief were dismissed as a low pain tolerance on more than one occasion.
I felt no one listened to me.
In both pregnancies, I said I needed to push, the midwives said I didnt (without checking) and in both cases I gave birth within quarter of an hour with only three or so pushes. In both cases I bled heavily afterwards

They did not believe me on when I told them that there had been multiple cases of stillborn and infection in my family.
This was my third birth and the midwife would not listen to me, go against textbook procedure/time constraints. My baby almost fell on to the floor as midwife was not ready. Pre-birth the consultant I was given was unsympathetic to the absolute agonising hell I was in
I felt heavily pressured to do what I was told rather than being allowed to trust my own body and instincts.
I didn't feel listened too or believed during my care, e.g., Effectiveness of pain relief or certain positions being painful. Also about my ability to push exhausting me and not progressing.
I think I was probably most frustrated by not feeling trusted to know my own body, especially for my second pregnancy in which the pelvic girdle pains were debilitating from very early on.
Thank goodness in my second pregnancy in 2015 the nursing staff listened to my concerns, they believed what I was saying and were really amazing. And I feel like if I hadn't been lucky enough to be at the hospital and asking for an ultrasound on a hunch that something wasn't right I would have lost my second pregnancy.
Second pregnancy I was prepared for the issues so care was better, but only because I fought my corner every step of the way.
I'm pretty knowledgeable about my health, I read up, and I'm confident enough to ask for what I need, so I was treated appropriately. I don't know how I would have been treated if I hadn't felt able to ask for things e.g., an anaesthetic assessment appointment in mid pregnancy and lots of extra monitoring.
Whilst hospital staff were caring and communicated well I was still not believed when I said the epidural was not working until I insisted they shielded my view and touched my toes, I then told them correctly which ones they touched.
I was not listened to regarding specific problems (hip pain, reasons for baby positioning, low blood pressure)
The supervisor of midwives was excellent and worked with me to have a very informative care/ birth plan which was more to educate the midwives rather than a typical birth plan.
Nurses, doctors, junior doctors were all very open and eager to learn about Ehlers-Danlos after meeting me on labour wards as to help other mums to be in my situation.
Patients with EDS etc know their own bodies well, what will help and what wont. We are at risk of serious permanent injury when medical professionals speak over us and dont listen to our needs.
I don't think the birthing team read the EDS print outs I provided on maternal care. Disappointed in that.
As both my pregnancies occurred before JHS diagnosis I was basically treated like a hypochondriac
Did not believe me when I had a very rapid labour. My mum had to find another midwife in the corridor to help deliver the baby.

	As a chronic pain sufferer I knew that the pain I was experiencing in early labour wasn't right, but I wasn't listened to and felt dismissed as a first time mum, who just couldn't handle pain.
	Dismissed anything myself or my husband had to say.
	It makes me sad to think back at how I constantly had to be my own advocate […] I have to constantly repeat that my knees HAVE to stay bent if I am under any anaesthesia to better keep them locked into place. I have spent my entire life protecting my knees
	When I went into labour it happened very quickly and no one believed me even though my care plan stated that this was likely
	My JHS was largely dismissed as a possible cause of issues: I was consultant led (UK) for other reasons but they had little interest in discussing my concerns and any issues my JHS may cause. I believe that it was part of the reason for my premature membrane rupture and then premature birth.
	Medical staff didn't seem bothered about the pain I was in.
	The hospital staff was not all well attuned to my needs, and I was lucky to have a stern midwife and husband to speak up for my needs.
	I had a very tough time no one taking me seriously until I ran into trouble and a shortening cervix was missed until I needed an emergency stitch. After that I was taken seriously, and my care transferred to a lead consultant.
	All staff listened and understood moving me had to be done carefully to avoid dislocations.
	I shared the latest research article with my midwife and this was passed to my consultant and added to the front of my notes. Subsequently I felt my care improved
	All support and care in relation to Ehlers-Danlos came from my information sharing.
	I found that I had to do own research and supply it.
	I am a registered nurse. I was well researched and advocated for the care I needed.
	I am also a midwife and fully understand my diagnosis and impact on pregnancy so informed my team and directed my care pathways.
	I am a registered midwife, so I had a good understanding regarding birth and the maternity system prior to my pregnancy
	I am also a midwife which I think influenced my choices
Maternity professionals	
	Details of patient experiences
	Already provide tertiary level mdt [multidisciplinary team] care in a maternal medicine setting, with the woman at the centre of her care (she is the expert)

	Time to spend with families so you can learn more
	That EDS presents differently in every woman, communication is key. It is essential to believe women when they report unusual symptoms, and not to fob them off because it doesn't fit in a box.
	Talking to the woman herself. She is the expert in her condition and not try and take away her choices. The woman I have supported at home, were all told they had to birth in hospital and be subject to interventions that were not necessary and not helpful. The more I read on EDS in maternity, the more I see control and coercion. None of this is helpful.
	Any information about how best to care for these women would be appreciated […] real life videos of women's experiences who have had babies with hEDS.
	[I was one of the only midwifery staff members who knew what the condition entailed…] she reported that she felt very safe with me as I already knew that her pain "was real" and "believed" her (which was the thing she found hardest in the maternity system reportedly - that people simply didn't believe her condition caused as much pain as it did).
	I have been involved in doing this [planning in conjunction with pregnant women with hEDS/HSD] for a few women now and I have learned a great deal from the women about their condition.
Subtheme 2: Coordinating appropriate perinatal care for people with hEDS/HSD	
Women with hEDS/HSD	
	I was offered morphine when it's in my notes I can't have it
	My midwife lost my notes, failed to arrange my first scan even after I had experienced a heavy bleed
	Hard to say as I never saw same MW twice
	I was supposed to labour gently because of heart issues but the Doctor sent away my anaesthetist
	I was not given the opportunity to see a anesthesiologist in my first pregnancy.
	My anaesthetist worked closely with my obstetrician to ensure an excellent delivery and briefed the theatre midwife appropriately.
	Very helpful and supportive physios to a GP who referred me to orthopaedics when I presented with severe PGP at around 7 weeks gestation (who in turn promptly sent me to physiotherapy)
	My maternity care was chaotic and poorly planned and executed leaving me feeling stressed about what was happening.
	The clinical references we provided 2 months in advance were not distributed to staff
	[I am a registered nurse] I built a team of skilled practitioners and had all appropriate interventions as necessary.
	I received physiotherapy which helped.

	Pregnant people with EDS need specialist care.
	It was known to various professionals (with my 2009 birth) that I was hypermobile due to previous dislocations. None of the maternity team were informed of this. Physiotherapist did not mention it throughout my care.
	Appropriate aftercare was significantly delayed (by years) and I had to push for investigation.
	I found it difficult to get the support I needed (and still do)
	In both my pregnancies I had a wonderful physio who relocated my pelvis on a weekly basis.
	Forceps delivery for my first son and then 3rd degree tears for my second all because the health care professionals would not allow me to give birth on all fours which would have enabled me to give birth easier (even though it was on my notes that I should birth in this position)
	My notes about EDS and my birth plan written by the doctors was ignored and led to complications
	My insurance was pregnancy only so they were unable to address my care even if they wanted to.
	Even though I had the benefit of private care, issues outside of "normal" pregnancy issues were dismissed, which reduced the quality of care & increased trauma
	The OB who was a geneticist was a strong advocate for me. She made all the difference with my medical team.
	Notes were not kept properly about my condition so every appointment I saw a different midwife or registrar who I had to explain everything again to.
	The number of different practitioners I saw for both pregnancies made it quite difficult to have any continuity of care.
	The care I had with the baby was excellent but no consideration was given to my lack of mobility and pelvic joint pain after the birth.
	I was pressured into accepting decisions that went again what the EDS specialist recommended and his views were completely disregarded
	The best care I had was from a specialist physiotherapist who put me in a hydrotherapy class for the SPD.
	I had very good care from my community midwife. She wrote in my notes what care I needed in labour to take into account that I had hypermobility syndrome.
	My maternal fetal medicine specialist […] knew all about EDS [...] my MFM always had my back and made sure she was in charge of my case.
	[No-one really understood EDS and] was bounced between Obstetric led care and Maternity care
	I wish that discharge included a referral for physical therapy post birth and that they routinely checked for dislocations.

	I strongly feel I should have been followed by a high-risk obstetrician not a midwife and that a lot of what happened could have been avoided.
	Had to give birth on back strapped to a bed despite pelvic pain and misalignment against physios wishes
	[my midwife did not know about my EDS], she referred me on to appropriate professionals immediately
	They [midwives] attempted to push forward my appointment with my consultant due to my joints deteriorating quickly in the third trimester however I was told by the consultant's secretary that this was not possible and went into late preterm labour following an attempt to relocate my hip myself at home.
	One Dr I seen knew about Hypermobility Syndrome, said I would need appointment with anaesthetist due to spinal problems and the fact I was getting induced. I never seen one!
	My diagnosis of HSD wasn't taken seriously or as a concern, but all of the symptoms of HSD I was experiencing were treated accordingly.
	I consulted my physio during my first pregnancy and they gave very helpful advice in managing symptoms and birth procedure, which was useful to aid later decisions.
	I was put in a group with non-hypermobile patients and taught exercises only one of which I could do because of my joints. They refused outright to take account of my inability, say, to be on my hands and knees, as they "only did lower back and pelvis", like I could somehow take all the other broken bits off and leave them outside.
	My final pregnancy was the only one I actually had a diagnosis. I informed everyone throughout, but they had me contracting for hours which is probably not the best thing to do with someone with a connective tissue disorder.
	Had I known that I had hEDS I feel that my care and experiences would have been very different for all fourth births as I would have been in a specialised birthing hospital.
	Services need to talk to each mother more so midwives are aware when mother comes in. There is no continuation in care.
Maternity professionals	
	Liaising with their existing care provider if they have been diagnosed, or a clear and open pathway for care outside of the maternity framework
	If there aren't enough staff you can provide all the information you like, the care will be ineffective.
	Good signposting when women present with conditions which are outside of our sphere of competence and knowledge, including which specialists to refer to locally.
	A small team of knowledgeable experts to support women through their hospital experience who update the staff regularly and take responsibility for the guideline.

	Individualised care depending on personal circumstances. Careful communication and planning
	Time to take a good history. Involvement and communication with the multidisciplinary team. Pre-existing health conditions to be diagnosed and a good management plan in place, including pre conceptual advice
	Anaesthetist who has experience in pain management
	Over 20 years of midwifery practice and I have had two cases only of what was called joint hyper-mobility. Both these cases were deemed by our doctors as mild but as a multidisciplinary team worked together to ensure best outcomes for both mother and infant on an individual bases.
	I believe it is important or these women to see the same midwife with an interest in EHD [sic] and associated conditions throughout the episode of care who can plan in conjunction with the woman for birth taking into consideration potential deterioration in condition.
Subtheme 3: Reducing trauma and improving experiences	
Women with hEDS/HSD	
	The midwives I had for my birth though were amazing !!
	[midwife knew nothing about EDS and had no interest] I didn't bother to arrange my last few appointments with them this pregnancy
	I feel far more disabled after having my baby. I'll never have another baby due to the poor treatment and care I received.
	Extremely traumatic experience as hEDS wasn't taken seriously by staff, and they ignored the fact that my baby was 4.5kg (exactly as predicted) leading to excess damage.
	The nurses were absolutely phenomenal and helped me so much. The doctor was not very good, she is the reason I tore so badly and she minimized my pain during the 6 months it took to heal
	Midwife was brilliant. But hospital care was not person centred, didn't follow birth plan and did not like patient choice (I had to quote policies and nice guidance). I felt that I was bullied into a hospital birth, a water birth was promised then when baby was coming they said it may not happen.
	Almost uniformly treated incredibly poorly, symptoms like high temperature and rapid pulse overlooked
	Terrible experience. No more babies for me.
	I was completely traumatised.
	I feel very angry and traumatised.
	I had a very pleasant experience with staff during birth and labour. During pregnancy, my care was very clinical and I felt somewhat dismissed by staff most of the time.
	Me and my husband made the tough choice to not have another child despite very much wanting to do so because of what we went through having my son.

	My first labour was extremely traumatic, physically and psychologically (the hospital involved issued a formal apology with regard to the treatment I received). It left me severe insomnia and post-natal depression, which was not picked up until 5 years later
	I was traumatised after I gave birth…the maternity carers were completely focused on the baby and not my mental health (due to the immense pain I was in).
	My local midwife was the most understanding and supportive.
	My first pregnancy I felt in very good hands. My second one, terrible.
	I had back-to-back contractions, they refused me pain relief and gas and air, and I had to labour in front of 3 other couples
	The maternity staff at the hospital where I give birth were horrible I was very unhappy and slightly traumatised by them and the birth. I am pregnant again and gone to a different hospital, I've received better care
	The midwives gave better care, listened more and were willing to research. The OBGYNs were unwilling and acted like I was malingering.
	First pregnancy the team were very over cautious and made many interventions that were probably unnecessary and caused further pain and distress
	I had no sympathy or help through my pregnancies as no one really understood. I was suicidal through my first one as no one understood, or believed, how agonising each day was.
	Difficult to rate above as 'maternity care' was so variable between community ante and post-natal, labour ward etc.
	Care with my midwife prior to hospital transfer was excellent. Care with the OB who performed my emergency caesarean was horrible, cold
	Hospital MW's [midwives] were excellent
	The care I received antenatally was variable…During my actual births/labour the care I received was excellent. But the care I received postnatally was terrible for both my pregnancies
	My lead maternity midwife was great. Care at hospital was not that good.
	My first pregnancy experience was not satisfying but my second was great
	I will never risk pregnancy and childbirth again because women with hEDS aren't given the healthcare services they need to ensure they aren't killed by the actions of ignorant medical and nursing staff.
	My experience with anaesthetists during labour has been nothing short of degrading and traumatic.
	Trauma. Just extreme trauma. And a sense I had been completely abandoned by the healthcare system during pregnancy with hEDS.

	I tore badly but refused to be swollen up due to midwife being awful and not wanting to be touched-trauma reaction not helped help due to pain relief being so ineffective during labour
	I did not die nor did my child apart from that fairly appalling.
	I was treated appallingly. I had a deeply unprofessional doula who refused to help me in crisis during a homebirth attempt, had to drive myself to hospital
	The care I received was excellent.
	In 5 pregnancies and 3 live vaginal births I have never been so surprised at the lack of staff care or support than I have around this most delicate time of a woman's life. I bruise my tailbone in labour and my minor labia is now completely torn and stretched from the original position. I feel mutilated and neglected.
	Lack of support around my own physical pain (other than the obvious of having a traumatic Orem [sic] birth) contributed to post-natal depression.
	The only problem with the second pregnancy was nursing staff unwilling to help at night when recovering from surgery. One nurse said that I'm going to have to take care of the baby at home, so I will need to stop asking for help.
	It's hard to answer this set of questions in a general sense, as I received different care for each of my three pregnancies. Some care I felt was better than others.
	My midwifery led home birth was amazing. The care was always respectful, evidence based and compassionate. I answered the above questions with my 2010 hospital birth in mind because it was so damaging physically and emotionally
	Each experience was very different for me, I know that I benefitted from the maternity care, with my second pregnancy I would have been in danger if it had progressed longer without intervention.
	After my experience I decided not to have any more children
	I would never agree to be pregnant again.
	I was treated appallingly. So badly that I refused to give birth in a hospital again.
	They made me feel guilty for getting pregnant in first place with all my difficulties.
	I was dropped by 5 doctors when they found out I was pregnant and told to terminate the pregnancy. I have a history of pulmonary emboli and needed daily blood thinner injections.
	After the baby was delivered and I had been stitched, a male surgeon shouted at me "Don't have any more children! No more!". His colleagues were shocked and just looked at me sympathetically. I felt so humiliated but because of the medication and how tired I felt, I could hear myself apologising and saying "ok". Afterwards I felt so angry that I didn't stand up for myself and it has stayed with me.
	PTSD- team were trauma informed and gave consideration to potential triggers

	They brought in students to watch because it was a difficult birth and I never approved that and I never would have allowed it. I was an unwilling spectacle. The look of horror on their faces at the trauma I was going through made it worse... It's been a complete nightmare and I'm scared for my own kids to have kids now.
	I had a very bad experience within my maternity care, I have since had a "debrief" with the family divisional HQ and found this to be patronising and rehearsed.
	Lack of care, dismissal.
	Awful experience
	[the local hadn't worked]. I was crying in pain and traumatised from my birth
	Whole experience has put me off having more children
	Although an earlier birth was a much more negative experience, this birth was still extremely hard, and I believe I was perceived as a difficult patient due to anxiety and pain. Maternity care did not seem to be at all flexible and any suggestions were shot down immediately.
	My Midwife was AMAZING and a total advocate for me. I found her during my second trimester after feeling very neglected and not taken seriously by my then Obstetrician.
	My actual care when I went into the hospital was rude and dismissive up until a midwife through from the labour ward actually spotted that I was basically ready to push
	I will never have another baby again because I don't have faith in maternity staff.
	The consultant at the hospital was awful and told me I had depression and not HSD, they also referred me to social services as I was unable to hold my baby post birth due to my shoulders constantly subluxating.
	It was an awful, frightening experience
	I begged for PT for pubic pain and they put down hip pain...I was in so much pain my 3rd trimester I was begging my husband to kill me
	Highly dismissive maternal care providers...Grade 4 placental abruption. Dismissed as a hypochondriac before medical providers clued into the severe blood loss
	I was put in stirrups and made to lie on my back in bed for over 24hrs. I was in agony
	The stitches I had following my first birth were excruciating and I really feared that I would need further stitches.
	I and the baby were neglected really as the hospital staff were kind but [they did not believe me on when I told them that there had been multiple cases of stillborn and infection in my family].
	I felt ignored during the birth, couldn't get what I needed
	Nurses ignored me when I told them not to bend my knee resulting in subluxation and retesting of prior knee surgery

	[I really don't think [obstetrician] knew anything about EDS or autoimmune disease, but then neither did I at that time]. They were still very supportive and made excellent decisions regarding my care. Just by listening, communicating well. We worked together and they were really proactive.
	The labour was awful and I was treated very badly.
	I remain angry [at] the damage that was done to my body and my mental health whilst under the care of midwives and physios during my two pregnancies, labour's, births and post-natal care.
	I received great care, but it was not necessarily the care I needed.
	I think EDS negatively impacted my pregnancy and especially my birth experience.
	If they had known about my hypermobility I would have hoped I would have been treated better.
	I am pregnant again and am terrified, have PTSD and cannot attend the hospital I birthed in. I will now be free birthing which isn't what I want but I refuse to be abused again
	I got pressured into various things I did not want. I was told I could have a waterbirth and last minute told I couldn't because of my EDS, a lady who had given birth the week before me had issues related to her EDS so therefore they didn't allow me to have a waterbirth even though i had no significant problems at all
	I had wonderful midwives & consultants
	I felt like my team knew what EDS type lll (my diagnosis) was but did not fully prepare me for possible complications. My 4th degree tear, which was blamed on the EDS, was therefore very shocking and has been something I have had to take time to process.
	The midwives who delivered my baby were extremely calming, welcoming, helpful and understanding. A couple of midwives in the aftercare made me feel quite silly regarding my hEDS, even though they didn't know what it was. The doctor who assessed my baby was very rude and dismissive about my hEDS and made me feel small emotionally.
	There is no excuse for the lack of help compassion and support for people in the medical profession. I still to this day sit without any regular support or therapy to fix what was done. No-one apologised to me and I feel angry at what has occurred.
	[Nobody had much experience if any of HMS] I was left struggling to walk, weak and exhausted. So much so it left a negative impact for me
	This meant the day before my baby was due I had to drive 40 minutes to the hospital by myself to have a scan. When I called to say my condition worsens if I am doing too much and I didn't want to drive to the scan I was told if I didn't go I would be referred to social services for not putting baby safety first. I found this ridiculous and showed the complete lack of knowledge or care for me and that the sole focus was my unborn baby.

	The nurses would realise baby hadn't been changed and did it for me. They didn't know about EDS. On one occasion, I was "told off" by a nurse because I was feeling dizzy after picking my baby up, trying to change his nappy when I finally remembered to, so I pressed the buzzer for help and she stormed in shouting at me and said "You are going to do this nappy yourself!". I was distressed and said I feel dizzy and am worried I'll drop the baby.
	Wouldn't want to do it again. Ever. [...] Stitches were also too tight which caused them to tear my skin during healing. Quite annoyed with that especially since I had asked them while they were stitching to follow the recommendations
	My care was incredibly substandard, and I probably wouldn't face half the issues I face today if they even had a tiny bit of knowledge or empathy towards this condition...spend each and every day suffering the impact of their mistakes and their dismissal of my concerns and my trying to make it known to them I have different needs to the norm which were consistently ignored. And as a result of their actions not only did I almost die, I have never been able to have the relationship with my son I desperately want to have, this has put a strain on my husband and my marriage. And it has also had financial consequences also due to my husband having to take leave to be mine and my sons carers. My care was incredibly substandard
	OBs and their nursing staff were universally the worst. They were dismissive, physically injured me repeatedly […] My midwives provided the vast majority of my care, however, and they were truly exceptional.
	I also get that my mental health was not prioritised.
	It was horrific and the worst experience of my life.
	Most importantly, I always felt in control of the process.
	I read about labouring in different positions to protect my hypermobile hip. I was physically prevented from trying these. I was forced into the lithotomy position and my legs were pressed down.
	I feel that the lack of care and understanding contributes to my PTSD and increasing anxiety and depression
	Although I received good care and support with my pain during pregnancy, I feel I could have been supported more with making informed choices about the birth. Staff didn't seem to acknowledge or validate my fears around natural birth and the potential strain and injury I may have sustained due to my JHS.
	It was very traumatic and as a nurse myself, it made me very angry.
	However [maternity professional] used scare tactics, told me I would bleed out if I had a home birth. Said I was high risk of haemorrhage. I had bleeding throughout the pregnancy requiring hospitalization twice but they never joined the dots with the EDS even when they determined the bleeding was to do with a cervical ectropion. I had a successful home birth using natal hypnotherapy techniques and felt much more in control the second time.
	I feel that the care for ladies with hEDS is completely inadequate.

	I do not feel that I was fully informed of my options or encouraged to explore them. I didn't want a caesarean, but I feel that was basically scared into having one.
	Because [my physicians weren't familiar with EDS], they were not properly prepared to aid in my delivery, which led to the separation of myself and my daughter.
	I disclose hypermobility and anxiety to midwife at 8 weeks. Neither was mentioned again.
	As I was high risk both times the level of care was poor. First pregnancy was shocking the treatment was terrible
	It [hEDS] wasn't taken into account during labour and positions during rapid birth contributed to prolapses afterwards
	No one knew I had EDS at the time and I hadn't been diagnosed yet with POTS, But they were good with my it mostly and treated it correctly despite me not having a diagnosis. They told me to do the same things my specialist told me once I finally got a diagnosis.
	My experience during pregnancy and childbirth meant I decided not to have any more than one child, even though I had wanted to have two children. There is no way I want to ever risk going through it all again. 11 years later and I'm still not over the trauma of the pain and lack of appropriate care and understanding from medical professionals.
	I told midwife about my back and small pelvis as I could feel baby stuck midwife said you knew you had a problem with your back and pelvis and you got pregnant now get this baby out
	But they were good with my it mostly and treated it correctly despite me not having a diagnosis. They told me to do the same things my specialist told me once I finally got a diagnosis.
	One of the reasons for my second pregnancy I am opting for a planned c section is because of how terrified I am of people not knowing how to help me
	I discussed my hypermobility with every maternity care professional I saw and was told I didn't need to worry. Unfortunately I ended up needing several operations to repair and re-attach due to severe pelvic region prolapse.
	Best practice in maternity care includes doing no harm and respecting patients. Sadly the high rate of birth trauma, mortality and birth injury evidences that there is still a significant way to go. Part of addressing EDS related maternity issues must include preventing diagnostic overshadowing and advocating for taking patient complaints seriously.
	The care and treatment from staff and Drs was disgusting I was dismissed as being an over anxious young mother

	Despite numerous warnings and meetings with staff we were pigeonholed as "anxious" and our concerns dismissed & warnings ignored.
Maternity professionals	
	Where are the women's input? What do they want? […] lumping all women together with the hEDS/EDS just gives them a label to be controlled by. I want to see more individualised care options and more input from women. Continuity of CARER models will start.
	I feel very few people know enough about it to provide personalized care to women who have the condition and so are at higher risk of not having the birth experience that they want or need.
	I have previously cared for women with Hypermobility, it was never named as Ehlers-Danlos Syndrome, I had difficulty getting obstetricians taking note when I was explaining my findings, they appeared to dismiss my concerns & plan of care.
	Women may be automatically considered high risk and not eligible for homebirth when it may well be the safest place to avoid birth before arrivals and interventions which may cause dislocations or inadequate pain relief.

Note. Square brackets provide additional context to aid interpretation for a quote within a theme. Quotes from maternity professionals were provided in responses to the following questions: "In general, what type of information would assist you in providing more effective care for all childbearing women in your role?" and "Please write anything else you would like to share with us below."
